# Efficacy of a smartphone application for helping individuals with type 2 diabetes mellitus manage their blood glucose: a protocol for factorial design trial

**DOI:** 10.1186/s13063-023-07489-5

**Published:** 2023-07-22

**Authors:** Hongxia Tang, Hua Qin, Mingjiao Zhang, Jihong Zhang, Huiwen Tan, Mengjie Chen, Laixi Kong, Maoting Guo, Fenghui Hu, Qin Wang, Xiaoxia Wang, Kun Zhang, Zhenzhen Xiong

**Affiliations:** 1 School of Nursing, Chengdu Medical College, No. 601 Tianhui Road, Rongdu Avenue, Chengdu, 610083 Sichuan China; 2 Hepatobiliary Vascular Surgery, The First Affiliated Hospital of Chengdu Medical College, Chengdu, 610500 Sichuan China; 3 Division of Endocrinology and Metabolism, The First Affiliated Hospital of Chengdu Medical College, Chengdu, 610500 Sichuan China; 4 West China Second University Hospital, Sichuan University, Chengdu, 610065 Sichuan China; 5 The School Hospital, Southwest Petroleum University, Chengdu, 610599 Sichuan China; 6 Division of Endocrinology and Metabolism, West China Hospital Sichuan University, Chengdu, 610000 Sichuan China; 7 Nursing Department, The First Affiliated Hospital of Chengdu Medical College, 278 Baoguang Avenue, Chengdu, 610500 Sichuan China

**Keywords:** Type 2 diabetes mellitus, Diabetes app, Factorial design trial, Chinese, Blood glucose management, Blood glucose monitoring

## Abstract

**Background:**

China has the largest number of individuals with type 2 diabetes mellitus (T2DM) in the world, and most lack knowledge about glycemic control and health management. This trial will examine whether a smartphone application can improve blood glucose management among individuals with T2DM.

**Methods:**

This will be a 2-center, factorial design, equal proportional distribution, superiority trial conducted in outpatient endocrinology clinics at two tertiary hospitals in Chengdu, China. The trial will enroll smartphone-literature individuals at least 18 years old who have been diagnosed with T2DM based on glycosylated hemoglobin (HbA_1c_) of at least 7.0%. Individuals will be randomly assigned to receive routine care with standard education about T2DM and glycemic control (Control), routine care as well as weekly telephone reminders to self-monitor blood glucose (Reminder), routine care and a smartphone application providing information about glycemic control and health management with T2DM (App), or the combination of routine care, the smartphone application, and weekly telephone reminders (App + Reminder). After 6 months of these interventions, participants will be analyzed for the primary outcome of HbA_1c_ as well as the secondary outcomes of blood glucose monitoring frequency, body mass index, blood pressure, knowledge about diabetes, health beliefs related to diabetes, diabetes self-management behavior, and satisfaction with the smartphone application.

**Discussion:**

This trial will determine whether a smartphone application can improve glycemic management among Chinese with T2DM. The findings may help guide the development of effective applications in China and elsewhere.

**Trial registration:**

Registration in the Chinese Clinical Trial Registry (ChiCTR) under registration number ChiCTR2100042297: https://www.chictr.org.cn/bin/userProject. 17 January 2021.

**Supplementary Information:**

The online version contains supplementary material available at 10.1186/s13063-023-07489-5.

## Administrative information

**Table:** 

Title{1}	Efficacy of a smartphone application for helping individuals with type 2 diabetes mellitus manage their blood glucose: a protocol for factorial design trial
Trial registration {2a and 2b}	Registration in the Chinese Clinical Trial Registry (ChiCTR) under the registration number ChiCTR2100042297: https://www.chictr.org.cn/bin/userProject
Protocol version {3}	Protocol version 3.0 (September 2021)
Funding {4}	This study is supported by Beijing Dnurse Technology Ltd (Beijing, China)
Author details {5a}	^a^ School of Nursing, Chengdu Medical College, Chengdu, Sichuan 610083, China ^b^ Hepatobiliary Vascular Surgery, The First Affiliated Hospital of Chengdu Medical College, Chengdu, Sichuan 610500, China ^c^ Division of Endocrinology and Metabolism,The First Affiliated Hospital of Chengdu Medical College, Chengdu, Sichuan 610500, China ^d^ West China Second University Hospital, Sichuan University, Chengdu, Sichuan 610065, China ^e^ The School Hospital, Southwest Petroleum University, Chengdu, Sichuan 610599, China ^f^ Division of Endocrinology and Metabolism, West China Hospital Sichuan University, Chengdu, Sichuan 610000, China ^g^ Nursing Department, The First Affiliated Hospital of Chengdu Medical College, Chengdu, Sichuan 610500, China
Name and contact information for the trial sponsor {5b}	DR Zhenzhen Xiong, Executive Director of Research, Chengdu Medical College
Role of sponsor {5c}	The funders played no part in study design, and will play no part in the collection, management, analysis, and interpretation of data, writing of the report, and the decision to submit the report for publication

## Introduction

### Background and rationale {6a}

In 2021, approximately 537 million people at 20–79 years old had diabetes, according to the International Diabetes Federation [1]. China contains the largest proportion of these individuals, most of whom have type 2 diabetes mellitus (T2DM) [1]. People with T2DM should self-monitor blood glucose levels in order to adjust their lifestyle and diet as needed to ensure good glycemic control. However, many individuals with T2DM do not monitor themselves [2, 3].

The ubiquity of smartphones and smartphone-based applications (“apps”) provides an excellent opportunity for educating individuals with T2DM about their condition and promoting self-monitoring of blood glucose. Current estimation suggests that there are nearly 1700 smartphone applications related to diabetes [4]. Studies so far have suggested that such applications can help people manage the disease [5] as well as reduce body weight [6] and blood glucose level [7].

However, a survey of 30 provinces in China in 2019 showed that only about 10% of individuals with T2DM used applications related to diabetes [8]. The frequency of using such applications was particularly low in Sichuan Province, which may reflect that its per capita gross domestic product (GDP) is substantially lower than that of the 10 Chinese provinces with highest per capita GDP [8].

A survey of more than 1276 individuals with T2DM identified “Dnurse” (dnurse.com/v2/app) as the most popular diabetes-related smartphone application in China [8]. The present trial will assess whether Dnurse can help individuals with T2DM manage their glucose levels and, more generally, their disease condition. It will also assess users’ satisfaction with the application.

### Objectives {7}

The aims of the study are to:

1. Evaluate the effect of the smartphone application Dnurse on glycemic management and disease control in individuals with T2DM
2. Assess whether the application influences compliance with blood glucose monitoring
3. Measure user satisfaction with the application

### Study design {8}

This will be a 2-center, factorial design, equal proportional distribution, superiority trial conducted in outpatient endocrinology clinics at two tertiary hospitals in Chengdu, China, which will be conducted from 2021 to 2023. 

**Fig. 1:**
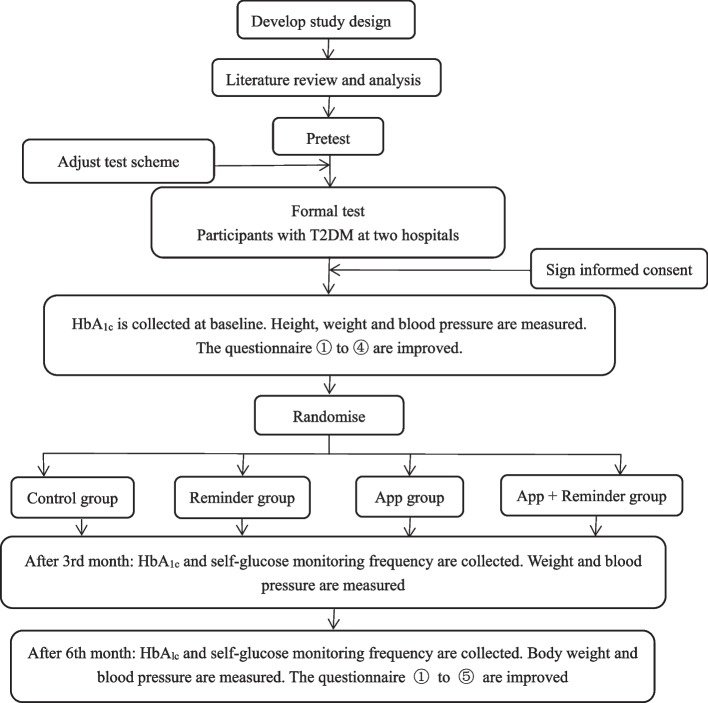
Flow diagram of the study. The questionnaires ① to ⑤ include the general situation questionnaire, Diabetes Knowledge Scale, Health Belief Questionnaire, Type 2 Diabetes Self-management Behavior Scale and User satisfaction with Dnurse application and its paired glucose meter

In reporting the protocol for this study, we followed the SPIRIT 2013 checklist. Individuals will be randomly allocated to the Control group, Reminder group, App group, and App + Reminder group. Flow diagram of the study is shown in Fig. 1.

## Methods: participants, interventions, and outcomes

### Study setting {9}

Since October 2021, the trial has been underway at two tertiary general hospitals (“grade A” in the Chinese hospital classification scheme) in Chengdu, China. At each site, two researchers have been designated to carry out the trial. A SPIRIT schedule of procedures and events is demonstrated in Table 1.

**Table 1:** Schedule of procedures and assess

**TIMEPOINT**		**STUDY PERIOD**		
	**Enrolement**	**Baseline**	**Intervention**	**Endpoint**
	**-**	**0 months**	**3 months**	**6 months**
**Enrolement:**				
Eligibility screen	**X**			
Informed consent	**X**			
Allocation	**X**			
**Interventions:**				
Control group		**X**	**X**	
Reminder group		**X**	**X**	
App group		**X**	**X**	
App + Reminder group		**X**	**X**	
**Assessments:**				
chronic complications related to diabete		**X**		**X**
acute complications related to diabetes		**X**		**X**
diabetes treatments		**X**		**X**
DKN, HBQ, 2-DSCS		**X**		**X**
satisfaction with the smartphone application				**X**
height, weight, blood pressure and HbA_1c_		**X**	**X**	**X**
frequency of blood glucose monitoring			**X**	**X**

Chronic complications related to diabetes: hypertension, cardiovascular disease, cerebrovascular disease, peripheral vascular disease, kidney disease, peripheral neuropathy, eye disease and diabetic foot; acute complications related to diabetes: hypoglycemia and lactic acidosis or ketoacidosis; diabetes treatments: non-drug therapy, oral hypoglycemic drugs, and insulin therapy

*DKN* Diabetes Knowledge Scale, *HBQ* Health Belief Questionnaire, *2-DSCS* Type 2 Diabetes Self-management Behavior Scale, *user satisfaction with Dnurse application*, custom-made questionnaire to assess user satisfaction with Dnurse application, HbA_1c_ Glycosylated hemoglobin

### Eligibility criteria {10}

Inclusion criteria are as follows:

1. Persons who are diagnosed with T2DM based on the criteria of the World Health Organization and diagnosed by a secondary or higher hospital
2. Persons glycosylated hemoglobin (HbA_1c_) ≥ 7.0%
3. Persons at least with a primary school education
4. Persons aged at least 18 years old
5. Persons with smartphone literacy. Smartphone literacy is assessed by asking potential participants whether more than three commonly used applications are installed on their smartphones
6. Persons who have residence in Chengdu for at least the previous 12 months
7. Persons who have willingness to participate and provide informed consent

Exclusion criteria include:

1. Persons with a previous diabetes-related application experience
2. Persons with a history of mental illness, cognitive impairment, or communication disorder, based on self-report
3. Persons with pregnancy or planning to become pregnant
4. Persons with any severe diseases posing threat to life
5. Persons with current diabetes-related hospitalization

### Who will take informed consent? {26a}

Written informed consent will be obtained from participants with type 2 diabetes participating in this study. Informed consent forms will be given to each potential participant, which they can read with their relatives and friends, and explanations will be given to help them make a decision. Participation in the study is completely voluntary, and participants can refuse to be in the study or withdraw from the study at any time without discrimination, medical treatment, and benefits will not be affected.

### Additional consent provisions for collection and use of participant data and biological specimens {26b}

The use of participants’ data and the collection of blood samples have been stated in the informed consent form, which have also been included in the ethics declaration.

## Interventions

### Intervention description {11a}

The Control group will undergo routine diabetes management: they will receive a standard portable glucose meter (Dnurse He pro wisdom; Beijing Dnurse Technology Ltd, Beijing, China) and blood glucose monitoring record book. The recommended timing and frequency of blood glucose monitoring will be explained to them according to national guidelines [9].

The Reminder group will receive the same materials as the Control group as well as a weekly telephone reminder to self-monitor blood glucose regularly. These reminders will be given by specially selected nursing undergraduates who have been trained in the rationale, interventions, and objectives of the study. Only nursing students who performed well on post-training testing are allowed to participate in the study. During the telephone call, students will ask participants whether they are self-monitoring regularly and, if not, what are the reasons.

The App group will receive a smart blood glucose meter (Dnurse SPUG mobile blood glucose uric acid tester; Beijing Dnurse Technology Ltd), and they will be led by a specially trainer researcher to download the Dnurse App 4.0.16 (dnurse.com/v2/app), designed to work smoothly with the smart blood glucose meter. The researcher will show the study participants how to open a personal account on the application and how to generate personalized diet and exercise programs, glucose control targets, and a blood glucose monitoring plan; how to access the food bank, disease information, and real-time blood glucose analysis and recommendations within the application; and how to set reminders to take medication and engage in other behaviors.

The App + Reminder group will receive the same materials and training as the App group, together with weekly telephone reminders like the Reminder group.

If members of the Control group and the Reminder group experience acute complications of diabetes, such as excessively high or low blood glucose, trial investigators will advise them to seek medical treatment as per standard procedures. If members of the App group and the App + Reminder group trigger a “critical value” alarm on the application, the customer service staff of the application’s manufacturer will call them by telephone and provide advice or information as per standard procedures. Both devices pass through ISO13485 and EU CE certifications.

### Criteria for discontinuing or modifying allocated interventions {11b}

Possible reasons for subject’s voluntary withdrawal are as follows: subjects found to be pregnant and subjects lost to follow-up.

### Plans to promote participant retention and complete follow-up {11c}

To promote participants’ participation in the trial, blood glucose test strips will be given to participants free of charge during the trial.

### Relevant concomitant care permitted or prohibited during the trial {11d}

Participants are allowed to go to the doctor or stay in the hospital normally during the trial, and the drug type and dose will be adjusted. If participants become pregnant during the trial, they will withdraw from the study.

### Provisions for post-trial care {30}

For the two groups that do not use the APP, we will introduce the relevant information of the Dnurse APP to them after the trial, and they will choose whether to use the APP for diabetes management according to their wishes.

### Outcomes {12}

Data are collected at three times, including two follow-up visits at baseline, the 3rd month after intervention, and the 6th month after intervention, through participant visits to the hospital by the investigator.

The primary outcome is the level of HbA_1c_, which reflects blood glucose levels over the preceding 2–3 months and is a good indicator of blood glucose control [10, 11]. All results will be made by high-performance liquid chromatography at the same certified external laboratory to ensure consistency.

Secondary outcomes include frequency of blood glucose monitoring, body mass index, blood pressure, knowledge about diabetes, health beliefs related to diabetes, and diabetes self-management behavior. The App and the App + Reminder groups will also be asked about their satisfaction with the smartphone application.

The following questionnaires will be used for the study:

1. Change in knowledge from baseline as measured by the Chinese version of the Diabetes Knowledge Questionnaire (DKN) [12]. Each item is scored from 0 to 1, and the total scores range from 0 to 23 (Higher score indicating greater knowledge of diabetes)
2. Change in health belief from baseline as measured by the Health Belief Questionnaire (HBQ) [13]. Scores ranging between 20 to 100 (higher score indicating better outcomes)
3. Change in self-management behavior from baseline as measured by the Type 2 Diabetes Self-management Behavior Scale (2-DSCS) [14]. Total scores range from 26 to 130 (higher score indicating better outcomes)
4. Custom-made questionnaire to assess user satisfaction with Dnurse application and its paired glucose meter. Participants who use the APP 6 months after the intervention will complete the assessment. The 5-level Likert scoring method is adopted, and the total scores range from 11 to 55 points (Table 2)

**Table 2:** Custom-made questionnaire to assess user satisfaction with Dnurse application

Item	Very satisfied	Satisfied	Neither satisfied nor dissatisfied	Slightly dissatisfied	Very dissatisfied
1. Your satisfaction with the application interface					
2. Your satisfaction with the application’s ease of use					
3. Your satisfaction with how the application records blood glucose					
4. Your satisfaction with the health information provided by the application					
5. Your satisfaction with using the application to manage diabetes					
6. Your satisfaction with the application’s safety					
7. Your satisfaction with the application’s performance (accuracy of results, speed of responses)					
8. Your overall satisfaction with the application					
9. Your satisfaction with the ease of operation of the glucose meter					
10. Your satisfaction with the stability of the connection between the glucose meter and application					
11. Your overall satisfaction with the glucose meter					

Instructions: Please choose the one response with which you agree most

### Participant timeline {13}

The SPIRIT participant timeline for the study can be seen in Table 1.

### Sample size {14}

Using GPower 3.1 (University of Kiel, Kiel, Germany), this experiment is a factorial design, and the parameters required for this experiment are not found in previous studies. Based on the expected results of the experiment, a small effect size of 0.2 [15], two-sided A = 0.05, test power (1-β) of 0.9, and the F-test are taken to calculate the sample size of 220 cases. To compensate for an estimated 20% loss to follow-up, the final total sample size is 264 cases.

### Recruitment {15}

Recruitment is carried out online, through announcements in outpatient departments, and by telephone from the National Center for Standardized Metabolic Management (Chengdu, China). For the online recruitment announcement, health education nurses at the First Affiliated Hospital of Chengdu Medical College have sent recruitment information to a WeChat group of individuals with T2DM that is managed by the National Center. Recruitment advertisements have been posted on the bulletin boards of outpatient departments at the two study sites. Researchers are also contacting participants using the telephone numbers on record at the National Center.

## Assignment of interventions: allocation

### Sequence generation {16a}

Before enrollment began, two sets of random numbers were generated using EXCEL by people not involved in the study. The serial number of each set was determined in advance and placed in an opaque, sequentially numbered envelope. Upon completion of the baseline assessment, eligible participants will be randomly (proportionally) assigned to one of four groups.

### Concealment mechanism {16b}

Before enrollment began, people not involved in the study used Microsoft Word to generate two sets of random numbers, determined the serial number of each group in advance and put them in sequentially numbered opaque envelopes, and hid them from all personnel, including the investigators, field staff, and participants.

### Implementation {16c}

Recruitment will be conducted by researchers and assisted by doctors and nurses at both hospitals’ diabetes clinics. After the baseline assessment is completed, each participant will select an envelope, which will indicate his or her allocation to one of the four groups described above.

## Assignment of interventions: blinding

### Who will be blinded {17a}

We do not blind the evaluators of the result scale and physical examination but only group them after baseline data collection to avoid the impact of subjective bias on baseline data. In the subsequent result evaluation, the evaluators of the scale and physical examination are not blinded, only the laboratory testers of HbA_1c_ are blinded, and the data analysis personnel are not blinded either.

### Procedure for unblinding if needed {17b}

The design is open label with only HbA_1c_ tester being blinded so unblinding will not occur.

## Data collection and management

### Plans for assessment and collection of outcomes {18a}

All participants will complete baseline assessments and complete follow-up at the 3rd and 6th month after the intervention.

At baseline, demographic data will be collected on age, sex, ethnicity, educational level, marital status, employment status, current residence, and method of paying for medical expenses. Data will be collected on the following clinical variables: chronic complications related to diabetes, such as hypertension, cardiovascular disease, cerebrovascular disease, peripheral vascular disease, kidney disease, peripheral neuropathy, eye disease and diabetic foot. Any chronic complications of diabetes that arise during the study will be recorded at the 6th month. Similarly, data on acute complications related to diabetes will be collected at baseline, such as hypoglycemia and lactic acidosis or ketoacidosis; and current diabetes treatments, including non-drug therapy, oral hypoglycemic drugs, and insulin therapy. Any acute complications of diabetes that arise during the study will be recorded at the 6th month.

At baseline and again at the 6th month, all participants will be asked to fill out three questionnaires. In addition, the App and the App + Reminder groups will fill out a fourth questionnaire at 6th month, which assesses their satisfaction with the smartphone application and glucose meter (Table 2). At baseline and again at the 3rd and 6th month, data will be collected on height, weight, blood pressure and HbA_1c_. At the 3rd and 6th month, all participants will be asked about their frequency of blood glucose monitoring.

### Plans to promote participant retention and complete follow-up {18b}

To promote participants’ participation in the trial, blood glucose meters will be given to participants who participate throughout the trial. We will provide certain transportation subsidies for participants who come to the hospital for follow-up visits.

### Data management {19}

The data will be managed in accordance with the requirements established by law. All data collected will be identifiable so that participants can be contacted. During the recruitment process, each participant is given a unique study identification number. Participants will complete an online questionnaire using the Questionnaire Star (https://www.wjx.cn/), and the data of the Questionnaire Star will be authenticated and recorded over a secure network connection. All other information about the participants will be stored in a secure, password-protected computer.

### Confidentiality {27}

Only researchers and project leaders have access to the data and may not disclose subjects’ personal information to third parties without authorization. Participants will be assigned a unique trial identification number and their details will be stored on a password-protected computer. Upon signing the informed consent, the subject will be informed that the information may be reviewed by the relevant national administrative authorities and the ethics committee, but any public reporting of the results of this study will not disclose the personal identities of participants.

### Plans for collection, laboratory evaluation, and storage of biological specimens for genetic or molecular analysis in this trial/future use {33}

This study is designed to collect HbA_1c_ specimens. If there are any remaining blood samples, the testing company shall dispose of them in accordance with Chinese laws and regulations.

## Statistical methods

### Statistical methods for primary and secondary outcomes {20a}

Data will be analyzed applying SPSS 23.0 (IBM, Chicago, IL, USA). Data will be expressed as mean ± standard deviation or as *n* (%) in the case of categorical data, and differences between the four groups will be assessed for significance using the chi-squared test in the case, analysis of variance in the case, or non-parametric rank-sum test in the case of skewed continuous data. Differences between different time points within each group will be assessed for significance using generalized estimation equation. These models will include intervention variables, time as a categorical variable represented by dummy variables, the interaction between intervention and time, and baseline values. Differences associated with *P* < 0.05 will be considered significant.

### Interim analyses {21b}

Interim analyses will be performed, and we will stop the trial based on interim data analysis, namely if clearly one treatment is better than the other.

### Methods for additional analyses (e.g., subgroup analyses) {20b}

We may analyze by comparing two study sites or educational levels.

### Methods in analysis to handle protocol non-adherence and any statistical methods to handle missing data {20c}

Data will be analyzed based on intentionality analysis. The extent to which data may be missing will be analyzed and examined, especially in view of the main findings of the study. Missing values are filled with the last data. For example, for participants without the 3rd month follow-up, the baseline data will be filled in. For participants with 3rd month follow-up but without 6th month follow-up, the 3rd month data will be filled in.

### Plans to give access to the full protocol, participant-level data, and statistical code {31c}

The datasets analyzed during the current study and statistical code are available from the corresponding author on reasonable request, as is the full protocol.

## Oversight and monitoring

### Composition of the coordinating center and trial steering committee {5d}

A trial steering committee will be established to manage, recruit, and ensure that the trial is conducted properly. The team consists of project leaders, researchers, and funders. A monthly meeting will be held in the trial preparation phase and a quarterly meeting in the trial conduct phase, and a written research report will be prepared to report the research progress and problems encountered. In addition, the School of Nursing of Chengdu Medical College and the Ethics Committee of the First Affiliated Hospital of Chengdu Medical College will supervise the study.

### Composition of data monitoring committee, its role and reporting structure {21a}

This is a low-risk intervention and Ethics Committee of the First Affiliated Hospital of Chengdu Medical College does not require one.

### Adverse event reporting and harms {22}

The adverse events in this study refer to hypoglycemia and will be registered at 3rd month and 6th month after the intervention. And the participants inform the doctor of the number of hypoglycemia times during outpatient visits.

### Frequency and plans for auditing trial conduct {23}

The trial review is conducted by two participating researchers at Chengdu Medical College from time to time during the recruitment and follow-up phases, including recruitment table, informed consent, compliance with the study protocol, and adverse events (if any).

### Plans for communicating important protocol amendments to relevant parties (e.g., trial participants, ethical committees) {25}

All relevant information will be shared among researchers. Important protocol modifications will be notified to the ethics committee and will be modified after being approved by the ethics committee. If a protocol modification may alter subjects’ perception of the risks or otherwise of the trial, this may lead to the requirement that investigators re-obtain informed consent.

### Dissemination plans {31a}

The results of this study will be published in a peer-reviewed journal, will be presented at national and international medical congresses, and also will be provided research reports to the project sponsors.

## Discussion

This trial aims to assess whether the smartphone application Dnurse can improve glycemic management and disease control in T2DM patients. Smartphone-based management and follow-up of diabetes is much less labor-intensive than direct follow-up by clinicians, and it can give individuals a sense of power and ownership of their condition, which may improve self-monitoring and treatment compliance [7, 16].

Despite the potential for smartphone applications to help people adopt healthier dietary and lifestyle behaviors [17], several studies suggest that they are not always effective. For example, a systematic review [18] found that only one of 11 studies assessing whether smartphone applications can improve T2DM self-management found a significant difference in HbA_1c_ related to application use, but none of seven studies assessing blood pressure found a significant difference, and only one of two studies assessing self-care found a significant difference. The investigators in those studies concluded that Apps had the potential to enhance diabetes management and glycemic control, but that further research was needed. The present trial will explore this potential using a rigorous, factorial design that will benchmark the application against routine care as well as telephone reminders. In addition, the trial will not exclude individuals with T2DM who are elderly or have diabetic eye diseases, which may make the findings of our study generalizable to a larger proportion of the global population with T2DM. Finally, our trial will substantially extend the literature on diabetes-related smartphone applications by examining a Chinese population, in contrast to the Western populations in most previous studies. China contains the largest number of smartphone users worldwide (nearly 1 billion), and the use of smartphone applications can be substantially higher than in the West [19].

In a survey in China, the Dnurse application was the most popular among T2DM patients and second most popular among individuals with type 1 diabetes [8]. It will be matched in the trial to a blood glucose meter from the same manufacturer, such that blood glucose data will be transmitted automatically to the application. This relieves the individual from the need of inputting data him- or herself, which may improve compliance with blood glucose monitoring [8].

## Limitations of the trial design

Although this trial will apply a rigorous factorial design to individuals recruited from two large medical centers in China, its design has some shortcomings that should be considered when interpreting the results. Whether the results obtained in this trial can be generalized to other T2DM, populations in China will need to be examined in additional studies. We cannot exclude that members of the Control group may use diabetes-related applications during the study, but at least they will be unable to use the Dnurse application, which is the focus of the trial. The follow-up period of 6 months will allow the effects of the application to be assessed in the mid-term while minimizing loss of participants, but longer follow-up will be needed to assess the application’s long-term effects, such as on the occurrence of diabetic complications.

## Trials status

The protocol is version 3.0 (September 2021). The trial commencement was in October 2021. We assume to complete the recruitment by the end of 2023.

## Supplementary Information

**Additional file 1.**

**Additional file 2.**

**Additional file 3.**

**Additional file 4.**

**Additional file 5.**

**Additional file 6.**

## Data Availability

The study co-primary investigators (Zhenzhen Xiong, Kun Zhang, Hongxia Tang and Hua Qin) have access to the final trial dataset, and any data required to support the protocol can be provided on request.

## Abbreviations

T2DM: Type 2 diabetes mellitus
HbA_1c_: Glycosylated hemoglobin
apps: Applications
GDP: Gross domestic product
DKN: Diabetes Knowledge Scale
HBQ: Health Belief Questionnaire
2-DSCS: Type 2 Diabetes Self-management Behavior Scale
