# Supplementary material for: Efficacy of a smartphone application for helping individuals with type 2 diabetes mellitus manage their blood glucose: a protocol for factorial design trial
Source: Trials. 2023 Jul 22;24:468. doi: 10.1186/s13063-023-07489-5 (PMC10362696; doi:10.1186/s13063-023-07489-5)
Supplement: Supplementary file 2 — Additional file 2. [file 13063_2023_7489_MOESM2_ESM.pdf]

[Editor's Note] Professor Weng Jianping, former director of the Diabetes Society of the Chinese Medical Association, pointed out that China has a vast territory, huge economic development level and diabetes treatment level in different regions, and the use of blood glucose monitoring is also uneven. On the one hand, the cross-sectional study of type 1 diabetes patients in Beijing and Shantou found that the average daily blood glucose monitoring of patients with type 1 diabetes was less than twice. In this regard, more education is needed to inform physicians and patients that effective blood glucose monitoring and treatment strategy adjustment can reduce the risk of hypoglycemia and long-term complications, reduce death and disability caused by diabetes, and greatly reduce medical expenses. On the other hand, in primary hospitals, there is also a phenomenon that continuous blood glucose monitoring is adopted regardless of patients' blood glucose level and medication plan, but blood glucose control still cannot be improved. It should be noted that blood glucose monitoring aims to improve clinical behavior and outcomes, and it is not meaningful to record blood glucose data alone. Therefore, physicians should clearly understand the significance of the monitoring of different methods, adopt reasonable monitoring combination for the clinical problems to be solved, formulate systematic and standardized monitoring programs, and improve clinical decision-making according to the monitoring results. Blind, unplanned blood glucose measurement should be avoided, and the economic burden placed on patients caused by blindly pursuing new technologies and new methods should be avoided.

Chinese Guidelines for the Clinical Application of Blood Glucose Monitoring (2015 Edition)

Diabetes Society of Chinese Medical Association

doi :10 .3969/j .issn.1672-7851.2016.05.003

Glucose monitoring is an important part of diabetes management, and its results can help to evaluate the degree of glucose metabolism disorder in diabetic patients, formulate a reasonable hypoglycemic plan, and reflect the effect of hypoglycemic therapy and guide the adjustment of treatment plan. With the progress of science and technology, blood glucose monitoring technology has also developed rapidly, and blood glucose monitoring is more and more accurate, comprehensive, convenient and less painful. Current clinical blood glucose monitoring methods include capillary blood glucose monitoring using blood glucose meter, dynamic blood glucose monitoring (CGM) continuously monitoring 3d blood glucose, glyc albumin (GA) reflecting the average blood glucose level of 2 to 3 weeks, and hemoglobin 1 c reflecting the average blood glucose level of 2 to 3 months (HbA 1c) detection, etc.

Capillary blood glucose monitoring includes patient self-blood glucose monitoring (SMBG) and bedside rapid blood glucose testing (POCT) in hospital, which is the basic form of blood glucose monitoring; HbA 1c is the gold standard reflecting the long-term blood glucose control level; and CGM and GA reflect the recent blood glucose control level, which is an effective supplement to the above monitoring methods. In recent years, 1,5-dehydrated glucose alcohol (1,5-AG), which reflects the blood glucose situation within 1 to 2 weeks, has also been gradually applied in clinical practice. In order to standardize the behavior of diabetes diagnosis and treatment, strengthen the effective management of diabetes, diabetes association in 2011 diabetes glucose monitoring group released in line with China's national conditions "China blood glucose monitoring clinical application guidelines (2011 edition)" (hereinafter referred to as the "guide"), but the present clinical medical staff attention to blood sugar monitoring and attention is still not enough, patients with diabetes

There is still a lack of guidance and education for blood glucose monitoring system, and some clinicians also lack of guidance and training according to the results of blood glucose monitoring and treatment behavior, so need to deepen the guidelines recommended blood glucose monitoring scheme, further training for medical staff, so as to better monitor the blood glucose level of patients with diabetes.

Capillary blood glucose monitoring

Capillary blood glucose monitoring including SMBG and POCT blood glucose monitoring in hospital two mode, it can reflect the real-time blood glucose level, assessment before meal, after hyperglycemia, life events (diet, exercise, mood, stress, etc.), and the influence of drugs on blood sugar, found hypoglycemia, help to develop individualized lifestyle intervention and optimize drug intervention program, improve the effectiveness and safety of treatment, is an important and basic daily management of diabetes patients.

S MBG

SMBG, as part of the self-management of diabetes, can help patients with diabetes to better understand their disease status, and provide an active participation in diabetes management, behavioral adjustment and drug intervention, and timely access to medical care

The means of worker consultation and thus improving compliance with treatment. Guidelines published by the International Diabetes Federation (IDF), the American Diabetes Society (ADA), and the National Institute for Health and Clinical Excellence (NICE) emphasize that the SMBG is a component of integrated diabetes management and education, and that SMBG is recommended for all diabetic patients to undergo SMBG. SMBG application in patients receiving insulin therapy can improve metabolic control and may reduce diabetes-related endpoint events, but the status of SMBG in the integrated management of diabetes in non-insulin-treated type 2 diabetes requires further study.

Blood glucose monitoring in the hospital

Hospital glucose monitoring can be measured in intravenous plasma or serum glucose by laboratory biochemistry instruments, but more blood glucose monitoring is done by rapid, simple and accurate POCT methods, so that patients can be treated accordingly as soon as possible. Countries for the medical institutions mainly in the ministry of health (now referred to as the development planning commission) of the medical institutions portable blood glucose detector management and clinical operation specification (WeiBan medical government hair [2010,209) as a guidance document, which made clear that blood glucose meter belongs to POCT equipment, its management should be as a POCT management of medical institutions

In part, the relevant rules and regulations for the clinical use and management of blood glucose meters should be established and improved, and the performance of hospital blood glucose meters is also required. It points out that not all blood glucose meters can meet the needs of hospital blood glucose monitoring.

The POCT method can only be used for monitoring blood glucose in diabetic patients and cannot be used for diagnosis.

Due to the situation of hospital patients is relatively complex, the patients of blood type, blood collection, blood hematocrit and various endogenous and exogenous substances on blood glucose detection, so for the hospital glucose meter accuracy and anti-interference, operator training and assessment, operating procedures and related system, quality control have more stringent requirements.

Scheme for capillary blood glucose

monitoring

1. Frequency and time point of blood glucose monitoring

The frequency and timing of blood glucose monitoring are determined according to the actual needs of the patient's condition. The frequency of blood glucose monitoring was selected at different time points of the day, including before meals, 2h after meals, before bed, and at night (generally from 2 to 3 am). The frequency of monitoring recommended by the domestic and foreign guidelines and the blood glucose monitoring at each time point

Table 1 Recommendations for the frequency of self-administered blood glucose monitoring (SMBG) for each guideline

therapeutic regimen	fingerpost	HbA 1c Failure (or treatment start)	HbA 1c Has reached the standard
insulinize	I DF (2012)	Most patients with type 1 diabetes mellitus and	
	CDS (2013)	pregnant women: 3 times / d	2~4 Times / d
		Admidia 5 times / d	
	ADA (2015) multiple injections or insulin pump therapy, SMBG time point: meals and	Before snacks, occasionally before meals, before bedtime, before transportation, before movement, when the hypoglycemia is suspected, treat hypoglycemia until after normalization, before performing key tasks (such as driving) SMBG results help guide treatment decisions and / or self-management	
Non-insulin	CDS (2013), 3d per week, 5 to 7 times / d		3d, twice /
	The ADA (2015) SMBG results help to guide treatment decisions and / or self-management		

Note: IDF, International Diabetes Federation; CDS, Diabetes Branch of Chinese Medical Association; ADA, American Diabetes Association.

Table 2 Scope of application of blood glucose monitoring at each time point

time	scope of application
Meprandial blood glucose	fasting blood glucose is high, or there is a risk of hypoglycemia (elderly, better blood glucose control)
2h postprandial blood glucose	fasting blood glucose has been well controlled, but HbA 1c [illegible]
	Patients with blood glucose injection before bedtime, especially patients with insulin injection before dinner
Overnight blood glucose	is close to the standard, but fasting blood glucose is still high; or [illegible]
	When other symptoms of hypoglycemia occur, blood glucose should be monitored in time, and blood glucose should be monitored before and after strenuous exercise

The scope of application is shown in Table 1 and Table 2.

2. Treatment principles of blood glucose monitoring

(1) Patients with diabetes control through lifestyle intervention can adjust their diet and exercise according to their needs to understand the effects of diet control and exercise on blood glucose through blood glucose monitoring.

(2) People using oral hypoglycemic drugs can monitor fasting blood glucose 2~4 times a week or 2 h after meals, or continuously for 3d within one week before the visit, and at 7 points a day (before breakfast, lunch, dinner and before bed).

(3) Blood glucose monitoring can be performed according to the insulin treatment protocol: ① Basal insulin fasting blood glucose should be monitored in patients using basal insulin, Adjust the dose of bedtime insulin according to fasting blood glucose; ② People using premixed insulin should be monitored for fasting and pre-dinner blood glucose, Adjust the pre-dinner insulin dose according to fasting blood glucose, Pre-breakfast insulin dose adjusted to pre-dinner glucose, If the fasting blood glucose reaches the standard, Pay attention to monitoring the postprandial blood glucose to optimize the treatment plan; ③ Meal-time insulin users should be monitored for post-meal or pre-meal blood glucose, The insulin dose before the previous meal was adjusted according to the postprandial blood glucose and the blood glucose before the next meal.

(4) The monitoring of special groups (perioperative patients, high-risk groups of hypoglycemia, critically ill patients, elderly patients, type 1 diabetes, gestational diabetes, etc.) should follow the above basic principles of blood glucose monitoring and measurement, and implement individualized

The monitoring scheme of the.

(5) For the goal of blood glucose control after monitoring, it is recommended to implement according to the comprehensive control objectives of the Guidelines for the Prevention and Treatment of Type 2 Diabetes in China (2013 edition). Some special groups (perioperative patients, hypoglycemia high-risk groups, critically ill patients, elderly patients, type 1 diabetes, etc.) can have loose blood glucose control standards, while gestational diabetes patients should strictly control the blood glucose level according to the Guidelines for Diagnosis and Treatment of Pregnancy with Diabetes in 2014.

3. The specific monitoring protocol is shown in the appendix.

Factors influencing capillary blood glucose monitoring

1. Accuracy factor of the blood glucose meter

The accuracy of the usual blood glucose meter includes two aspects: accuracy and accuracy. Accuracy is the degree of agreement between the measurements of the blood glucose meter and the laboratory blood glucose tests, and the degree of agreement after multiple repeated measurements of the same sample. At present, the standard of ISO 15197-2013 is followed internationally.

Accuracy requirement: The deviation between the whole blood results and the plasma results of the same site should be controlled in the following range: at least 95% of the test results are met, when the blood glucose concentration is <5 mmol / L, it should be within the ± 0 mmol / L deviation; when the blood glucose.6.83

.6At a concentration of 5 mmol / L, it shall be within the ± 15% deviation.99% of the resulting bias was within the clinically acceptable range for the concordance network error analysis grid.

Precision requirement: at blood glucose concentration <5 mmol / L, standard deviation <0 mmol / L; blood glucose concentration 5 mmol / L, coefficient of variation (CV) <7.5%。.6.42.6

2. Interference factors

At present, the clinical glucose meter detection technology adopts biological enzyme method, mainly including glucose oxidase (GOD) and glucose dehydrogenase (GDH), while GDH also requires different coenzymes, namely pyrroquinone glucose dehydrogenase (PQQ-GDH), flavin adenine dinucleotide glucose dehydrogenase (FAD-GDH) and nicotinamide adenine dinucleotide glucose dehydrogenase (NAD-GDH). The GOD glucose meter is highly specific for glucose and is not disturbed by other sugars, but is susceptible to oxygen interference. The GDH glucose meter does not require the participation of oxygen and is not disturbed by oxygen. The glucose meter of FAD-GDH and NAD-GDH principle cannot distinguish xylose from glucose, and the glucose meter of PQQ-GDH principle cannot distinguish maltose, galactose and xylose from glucose, which may react with maltotose and galactose in blood samples, leading to the false elevation of blood glucose results. The mutation-modified Mut. Q-GDH principle of blood glucose meter no maltose, xylose and other sugar substance interference.

The blood samples used by the blood glucose meter are mostly whole blood, so the hematocrit is greatly affected. In the same plasma glucose level, with the increase of hematocrit, the detection value of whole blood glucose will gradually decrease. This difference was minimized by glucose with hematocrit correction. Common disturbances are: acetaminophen, vitamin C, salicylic acid, uric acid, bilirubin, triglyceride and other endogenous and exogenous substances. When there are a large number of distractions in the blood, the blood sugar value will have a certain deviation. PH, Temperature, humidity and altitude are necessary for the optimal operation of the glucose meters and dipstick.

3. Factors of the difference between capillary blood glucose and venous blood glucose

Usually the blood glucose meter uses capillary whole blood, while the laboratory tests are venous serum or plasma glucose, with plasma calibrated blood glucose meter tests

The fasting value is close to the laboratory value, and capillary glucose will be slightly higher than venous blood glucose after eating or after taking glucose. If the whole blood calibrated blood glucose meter is used to detect the fasting value is about 12% lower than the laboratory value, capillary glucose is close to venous plasma blood glucose after eating or after taking glucose.

4. Operator's technical factors

Improper operation, insufficient blood volume, local extrusion, replacement of test paper batch number correction code or improper preservation of test paper will affect the accuracy of blood glucose monitoring.

Patient education

Patient education includes standardized blood glucose test and records, the interpretation of blood glucose results and how to make diabetes education with diabetes patients realize the importance of blood glucose monitoring, blood glucose test results itself to the improvement of the disease, need medical staff and patients to discuss the results of blood glucose test and take measures to actively change individual behavior and adjust the treatment plan, to make blood glucose monitoring become effective diabetes self-management tool.

1. Blood glucose testing and recording

In the actual patient self-monitoring process, the user's operation technology is also a key factor affecting the accuracy of blood glucose measurement results. The following three steps can regulate the patient's operation.

(1) Preparation before test: Prepare blood collection tools, blood glucose meter and blood glucose test paper, operate in strict accordance with the operation instructions of blood glucose meter and measure them within the appropriate operating temperature range of blood glucose meter; clean the blood collection site (such as the side of the abdomen) and handle with soap and warm water (Especially the blood collection site) Wash and dry with a clean napkin or cotton ball; after cleaning, drop the arm for a moment, then massage the blood collection site and use the appropriate blood collector to obtain sufficient blood samples. Do not squeeze the blood sample, otherwise the tissue will dilute the blood sample and interfere with the blood glucose test results.

(2) Requirements in the test: it is recommended to absorb sufficient blood samples at one time (some blood glucose meters that meet the secondary sample design); do not press or move the blood glucose test and blood glucose meter during the test.

Diabetes World • Clinical, Volume 10,2016

(3) Post-test requirements: record the blood glucose test results. If the test results are suspicious, it is recommended to test them again once. If there is still any doubt, consult the medical staff or contact the glucose meter manufacturer. Before determining the cause and consulting the medical staff, remove the blood glucose strip and discard it in the appropriate container with the needle; store the blood glucose test supplies (blood glucose meter, blood glucose strip, blood collector, etc.) in a dry cleaning place.

2. Quality and quantity control

After replacing the new blood glucose meter, the new test strip and the battery, the instrument should be corrected with the random simulation liquid or quality control liquid. When the capillary blood glucose results are inconsistent with HbA 1c or clinical situation, or the blood glucose meter is inaccurate, the instrument should be calibrated at any time.

3. Management of capillary blood glucose data

Blood glucose log should contain various information such as blood glucose, diet, exercise, conditional computerized data management, using USB, or wireless transmission technology to connect blood glucose meter with a computer, download blood glucose data with blood glucose management software, can display blood glucose records, blood glucose trend chart, 14d atlas, etc., can be better used to evaluate blood glucose control trend and the influence of drugs, diet and exercise on blood glucose control, and guide the optimization of treatment plan. As a new medical method, mobile health care rationally allocates medical resources through information technology and improves the utilization rate of medical resources, so it has received more and more attention in the field of diabetes management. Mobile health care provides medical services and information through mobile communication technology. At present, it mainly provides short messages and medical applications (apps) based on mobile terminals such as Android (Android) and Apple (IOS). Mobile health care mainly has short messages and smart phone App in diabetes management. Both short message and smartphone App can record patients 'blood glucose monitoring status. Previous studies have shown that it can promote patients' lifestyle adjustment, improve patients' blood glucose control, optimize hypoglycemic treatment plan, and realize individualized diabetes management. App has been approved by the Food and Drug Administration (FDA) as a medical device for diabetes. At present, there is still a lack of clear laws and regulations for mobile health care in China, and its application in diabetes management needs to be further explored.

4. Guide the patients

Inform patients of self-diabetes management of glucose control goals and the purpose of monitoring, guide patients on how to interpret the monitoring results and how to take action with reference to the results. At the same time, the medical staff should carefully review the blood glucose records and adjust the treatment plan according to the blood glucose monitoring results.

Limitations of the capillary blood glucose

Due to the limitation of blood glucose meter detection technique and blood collection site, capillary blood glucose has some limitations: poor local circulation, such as shock, severe hypotension, diabetic ketoacidosis, diabetic hyperotonic coma, severe dehydration and edema, no capillary blood glucose detection; needle sampling may cause discomfort; non-standard operation may affect the accuracy of blood glucose measurement results; when the monitoring frequency is insufficient, the judgment of average blood glucose, blood glucose fluctuation or incidence of hypoglycemia should be cautious; too frequent monitoring may lead to anxiety in some patients.

HbA 1c

HbA 1c Is an indicator reflecting the average blood glucose level of the previous 2 to 3 months. It has been used as the gold standard for evaluating long-term blood glucose control clinically, and also an important basis for clinical decision whether to adjust treatment. Large clinical trials such as the Diabetes Control and Complications Study (DCCT) and the UK Prospective Diabetes Study (UKPDS) on type 2 diabetes have demonstrated that intensive glycemic control targeting HbA 1c reduces the risk of microvascular and macrovascular complications in diabetes.

Detection method of HbA 1c

According to the different detection principles, the determination method of HbA 1c is divided into two categories. One is to distinguish A 0 and A 1c according to the different charge of hemoglobin (Hb), including ion exchange high performance liquid chromatography, capillary electrophoresis method and isoelectric focusing method; the other is to use the molecular structure difference between HbA 1c and non-HbA 1c, using affinity chromatography and immunochemical method. At present, the most commonly used detection method is high performance liquid phase, which has high precision, repetitive and simple operation, and has been widely used in clinical practice.

HbA 1c Standardization of the assays

Since the description of HbA 1c in the late 19 60s, multiple HbA 1c tests have been established due to the diversity of biochemistry, and clinical needs of glycosylation and management. Although the issue of standardized HbA 1c determination was raised as early as 1984, it did not begin to receive attention until after the results of the DCCT were published in 1993. In 1993, the American Society of Clinical Chemistry (AACC) established a sub-committee to conduct the standardization of HbA 1c determination, so that the results of different methods can be traced to the reference results of DCCT. In 1996, the standardization work was completed by the US National HbA 1c Standardization Program (NGSP) Steering Committee. In 2000,90% of laboratories in the United States reported hemoglobin results as HbA 1c, and all participating NGSP laboratories were <5%, and the deviation of HbA 1c results from the target value was <0.8 %。The US Committee on Clinical Laboratory Standards (NCCLS) adopted the standardized model of HbA 1c measurement by NGSP. Although HbA 1c standardization started late, it developed rapidly. In 2010, led by the Science Popularization Department of China Association for Science and Technology and the Science Popularization Department of Chinese Medical Association, the Clinical Laboratory Management Committee of Chinese Hospital Association launched the "China HbA 1c Education Plan" as the cooperative unit. Subsequently, the State Food and Drug Administration issued the "HbA 1 c Analyzer" pharmaceutical industry standard. In 2013, the Clinical Laboratory Center of the National Health and Family Planning Commission officially published the Guidelines for Laboratory Testing of Hemoglobin A 1 c, which regulated the analysis of interference factors, selection of methods, use of methods and quality monitoring of measurement results in HbA 1c testing. The number of laboratories participating in the inter-room quality evaluation program organized by the National Clinical Laboratory Center has increased year by year, from more than 20 in 2000 to more than 800 in 2012. The coefficient of variation of HbA 1c detection decreased annually from 20% to 30% to 4% in 2012.6%~5 .3% 。The standardization of HbA 1c has laid a good foundation for its further clinical application in diabetes prevention and treatment in China.

In 1 9 9 5, the International Alliance for Clinical Chemistry and Laboratory Medicine (IFCC) A dedicated working group was established to develop standard materials and standard methods for HbA 1c determination and to study a reference system for traceability. In its established laboratory network, it was calibrated using purified HbA 1c and rapid hemoglobin (HbA 0) and worked to establish links with the NGSP results. The experimental results showed that the HbA 1c results of IFCC were slightly lower than the NGSP results, but the experimental operation was complicated,

It takes a long time and has a high cost, and should not be used as a routine clinical examination. During the "11th Five-Year Plan" period, the Clinical Laboratory Center of the Ministry of Health (now the National Health and Family Planning Commission) established the HbA 1c first-level reference method (IFCC HPLC-LC-MS / MS), developed three levels of national first-level standard materials, and carried out HbA 1c correctness verification nationwide.besides, The IFCC HbA 1c primary reference laboratory established by Shanghai Clinical Laboratory Center passed two comparison studies in 2012 in July and October 2012 respectively, Received the 2013 annual certificate issued by the IFCC HbA 1c Network, In 2013, the compartment quality review officially launched the "glycated hemoglobin correctness verification plan", Values were assigned to the correct samples using the IFCC HbA 1c first-level reference method, When the results of different hospitals, different equipment and different methods are compared with a correct value, To evaluate the accuracy of their results.

Based on the above background, we suggest that: (1) at present, standardized HbA 1c results should be used to estimate the average blood glucose level; (2) Participate in the interroom quality assessment of the health administration department; (3) HbA 1c is still a key indicator of diabetes management, so the change of HbA 1c reference range should be avoided as far as possible.

The clinical application of HbA 1c

1 to assess the glycemic control status in diabetic patients

According to the Guidelines for the Prevention and Treatment of Type 2 Diabetes in China (2013 edition), the test should be conducted at least once every 3 months at the beginning of treatment, and once every 6 months once the treatment target is reached. HbA 1c The determination shall be performed using a method traceable to that previously used by DCCT. The control objectives of HbA 1c for type 2 diabetes are detailed in the Guidelines for the Prevention and Treatment of Type 2 Diabetes in China (2013 edition).

2. Diagnosis of diabetes mellitus

In the past, because the detection of HbA 1c was not standardized enough, so it was not recommended for the diagnosis of diabetes mellitus. In recent years, standardized testing of HbA 1c has been improved globally, especially since a new and more specific testing reference was established in 2003, facilitating the reevaluation of HbA 1c as a method for diabetes screening and diagnosis.

In 2009, by ADA, European Diabetes Society (EASD) and IDF

The international expert committee, in reviewing from Egypt, Pima Indian, the basis of a application of HbA 1c for diabetes diagnosis report, said that using HbA 1c 6.5% to identify patients at risk of progressive retinopathy, and thus make diabetes diagnosis, has sufficient sensitivity and specificity. In 2010, ADA will HbA 1c 6.5% were included in the diagnostic criteria for diabetes mellitus. In 2011, WHO officially released the consultation report of "Using HbA 1c to diagnose diabetes", which recommended HbA 1c testing as an auxiliary means for the diagnosis of diabetes where conditions permit, and 6.5% was the cut-off value for the diagnosis of diabetes. Meanwhile, HbA 1c <6.5% could not rule out diabetes diagnosed by blood glucose testing. Due to ethnic differences in HbA 1c, the cut-point of HbA 1c diagnosis in Chinese population may be different from foreign criteria. In recent years, domestic scholars have also carried out corresponding studies to explore the value of HbA 1c screening and diagnosis of diabetes and pre-diabetes in Chinese population. The results of several community-based HbA 1c and oral glucose tolerance test (OGTT) and community-based cross-sectional studies of HbA 1c and retinopathy suggest that the best cut-point for HbA 1c diagnosis of diabetes in Chinese adults is 6.2%~6 .4%, with 6.3% of the evidence was more, lower than ADA and WHO, published HbA 1c 6. And 5% of the diagnostic criteria for diabetes mellitus. However, in view of the fact that HbA 1c testing is not common enough in China, and the standardization degree of testing methods is not high enough, the instrument and quality control of HbA 1c measurement can not meet the current requirements of diabetes diagnosis, so it is still necessary to further promote the process of HbA 1c testing standardization, so as to facilitate the application of HbA 1c to diagnose diabetes.

Advantages of the HbA 1c detection

1. There is no need for an empty stomach for patients, and blood samples can be collected at any time without affecting meals.

2. Compared with venous blood glucose, it can better reflect long-term blood glucose, and is not affected by short-term diet, exercise and other lifestyle changes.

3. HbA 1c laboratory test methods are being standardized.

4. The errors caused by some non-glycemic factors affecting HbA 1c are rare, such as hemoglobinopathy.

Diabetes World • Clinical, Volume 10,2016

Factors affecting HbA 1c test

results 1. Effect of the renewal

rate of hemoglobin on HbA 1c values

Any factor that can cause an increase in the average lifespan of red blood cells will increase the concentration of HbA 1c independent of blood glucose levels, such as a decrease in erythrocyte clearance after splenectomy.

Any factor that may shorten the life of red blood cells may reduce HbA 1c, such as hemolytic anemia, because hemoglobin binds less with surrounding glucose in immature red blood cells and active bleeding can increase the generation of reticulocytes, thereby reducing the average life of red blood cells in uremic patients on dialysis.

2. Drugs

Vitamin C, vitamin E, high doses of salicylate, erythropoietin treatment, antiretroviral drugs, ribavirin and dapsone can reduce the measurement results.

3. Ethnic differences

HbA 1c Ethnic differences are present and are independent of blood glucose levels. Diabetes Prevention Study (DPP) and Diabetes outcome Progress Study (ADOPT) Black Americans had a higher HbA 1c than Caucasians 0.4% ~0 .7%。The extent of HbA 1c differences among different ethnic groups should be further investigated.

4. Sample storage time and temperature

The results can increase gradually with the storage time. The ion-exchange chromatography is relatively well stable at any temperature. Most test samples can be stored at-70℃ for 1 year, and whole blood samples can be stored at 4℃ for 1 week for only a few days at room temperature. Untreated whole blood samples were poorly stable and all had effective storage times less than 1d.

5. Some disease states

Hypertriglyceridemia and hyperbilirubinemia can raise the HbA 1c water

Flat, while chronic liver disease can reduce HbA 1c levels.

6. Pregnancy

The HbA 1c level was slightly lower in women in the second trimester and slightly higher in the third trimester.

Limitations of the HbA 1c

The test results had a "delayed effect" on the assessment after adjusted treatment, which did not accurately reflect the risk of hypoglycemia or the characteristics of blood glucose fluctuations.

GA

Glycated serum protein (GSP) is the product of a non-enzymatic reaction between blood glucose and protein (about 70% albumin). The binding process of various serum proteins to sugars is essentially identical, and the nonionic ε -or α -amino groups on the protein molecules form unstable compounds with the carboxyl group on the aldose, namely the Schiff base. This is a reversible reaction, Schiff base can dissociate into protein and aldose, and can generate more stable ketamine through translocation rearrangement. Its structure is similar to fructosamine (FA), so the GSP determination is also called fructosamine determination. Since albumin has a short half-life in vivo, from 17 to 19 d, the GSP level can reflect the mean blood glucose level from 2 to 3 weeks before detection in diabetic patients. The GSP measurement method is simple, time-saving and does not need special equipment, and can be widely used in primary medical units. However, because GSP measurement reflects the total glycosylated plasma protein in plasma, its value is susceptible to the influence of blood protein concentration, bilirubin, chyle and low molecular substance, especially in patients with hypoproteinemia and abnormal albumin conversion. Meanwhile, due to nonspecific reducing substance in serum, and different non-enzymatic glycoreaction rate of different protein components, GSP detection method is poorly specific and is gradually replaced by GA.

GA is a quantitative measurement on the basis of GSP, which uses the percentage of serum GA and serum albumin to express the level of GA. The effect of serum albumin level on the test results is removed, so it is more accurate than GSP. In recent years, it has been gradually popularized in clinical practice.

The detection method of the GA A

The earliest GA measurement was the high-pressure liquid phase ion exchange method (HPLC method) developed by Japanese scholars, but this method has a small sample size, which is not suitable for routine clinical practice and has not been widely used. In 2002, the United States developed a solid enzyme method, with high specificity, but for patients infused with high energy amino acids, the measurement results will be abnormally elevated. In recent years, the enzymatic detection of GA (GA-L) developed in Japan has good dilution linear performance, intraday repeatability and daytime stability, and has good consistency with HPLC detection method, so it is the most used in clinical practice. Since 2003, the liquid enzyme method has been studied in China and applied in clinical practice. In 2005, Yamaguchi et al. reported a detection system for GA measurement by dry enzyme method. The detector requires a small amount of blood samples, and can measure the GA value within 5min, which has a good correlation with GA-L. From the HPLC method, the solid enzyme method, to the recent liquid enzyme method, and even the recent dry enzyme method, the GA detection method gradually tends to be simple, fast, accurate and practical. Moreover, GA-L can be tested on any automatic biochemical analyzer, making its widespread application in clinical practice possible.

Normal reference values for the GA

As a new monitoring method, GA is lacking a recognized normal value due to its relatively short time in clinical practice. In recent years, studies on GA normal reference value have been carried out in China. In 2009, Shanghai Diabetes Institute adopted the clinical collaborative study of 10 centers nationwide, and 380 normal people aged 20-69 were selected and the normal reference value of Chinese GA was 10.8％~17 .1％。During the same period, the study in Beijing area showed that the normal reference value of GA was 11.9%~16.9%.

The clinical use of the GA

1. Evaluation of the short-term glucose metabolism control situation

GA is sensitive to blood glucose changes in the short term than HbA 1c, it is usually believed that GA can reflect the average blood glucose level in nearly 2~3 weeks, is a good evaluation of short-term glucose metabolism control index, especially for diabetes patients after the treatment of evaluation, such as short-term hospitalization of diabetes patients, GA may have more clinical reference value than HbA 1c.

In addition, GA can assist in the identification of acute stress-induced hyperglycemia, such as trauma, infection, and acute cardiovascular and cerebrovascular events. The combined measurement of GA and HbA 1c is helpful to determine the duration of hyperglycemia, and can be used as an auxiliary test for previous diabetes mellitus.

2. Screening for diabetes mellitus

GA is also suitable for the screening of diabetes, and most undiagnosed diabetes patients can be screened out in GA 17.1%. Abnormal GA is an important indication indicating that people at high risk of diabetes should undergo OGTT examination, especially for those with normal fasting glucose. Of course, further prospective epidemiological studies are still needed on whether GA can be used as an indicator for diabetes screening.

3.GA and diabetes mellitus complications

There has been evidence that GA, as an important glycosylation product, is correlated well with chronic complications such as diabetic nephropathy, retinopathy and atherosclerosis.

Advantages of the GA assay

For patients with diabetes who affects red blood cell life span, HbA 1c measurement is often underestimated, when GA measurement is not affected. Therefore, GA is more reflective of glycemic control than HbA 1c.

Factors influencing the results of the GA

testing test

1. Effect of the renewal speed of blood albumin on the GA results

The rate of blood albumin affects the level of GA values. For the same blood glucose levels, individuals with accelerated blood albumin renewal had lower GA levels. Therefore, this factor should be taken into account when assessing the levels of GA in diabetic patients with abnormal albumin transformation (e. g., nephrotic syndrome, abnormal thyroid function, cirrhosis).

2. Body, fat content

Physical fitness index (BMI) is an important factor affecting GA level, which has a negative effect. The reasons are not clear, which may be related to the speed of albumin renewal, catabolism and inflammation in obese people.this

Besides, the negative effect of increased body fat on GA levels may act mainly through fat mass and intra-abdominal fat. Therefore, GA may underestimate their actual blood glucose levels in people with increased body fat content or central obesity.

3. Thyroid hormone

Thyroid hormones can promote the breakdown of albumin, which can also affect serum GA levels. Hyperthyroidism can reduce the results and hypothyroidism.

Limitations of the GA detection

Compared with HbA 1c, GA reflects the level of glycemic control in a short period of time, and there is a lack of large sample and prospective studies on GA and chronic complications of diabetes. Therefore, GA should be cautious in clinical monitoring of long-term glycemic control level. GA does not reflect the characteristics of blood glucose fluctuations.

1,5-AG

1,5-AG is the C-1 deoxygenation form of furan glucose, and its content is second only to glucose in polyols. It is significantly reduced in diabetic patients, which can accurately and quickly reflect the blood glucose control within 1 to 2 weeks, especially for the monitoring of postprandial blood glucose fluctuations. In 2003, the US Food and Drug Administration (FDA) approved 1,5-AG as a new indicator to evaluate short-term blood glucose monitoring. It has been shown that 1,5-AG can be used as adjunct to guide modification in the treatment of diabetes management. However, the significance of 1,5-AG in diabetes screening and diagnosis needs to be confirmed by more evidence-based medical evidence.

C GM

CGM refers to the monitoring technology that indirectly reflects blood glucose level by monitoring glucose concentration by glucose sensor. It can provide continuous, comprehensive and reliable blood glucose information throughout the day, understand the trend of blood glucose fluctuations, and find hidden hyperglycemia and hypoglycemia that are not easy to be detected by traditional monitoring methods. Therefore, CGM could be one of the traditional blood glucose monitoring methods

Effective supplement. The CGM techniques are divided into two types: retrospective and real-time CGM. Clinical studies conducted at home and abroad show that both retrospective and real-time CGM have good accuracy and safety. The clinical advantages of CGM technology, indications, accuracy evaluation of monitoring data, interpretation of monitoring results and how to guide clinical practice are introduced in detail in the Guidelines for Clinical Application of Dynamic Blood Glucose Monitoring in China, among which the following points should be paid special attention to.

Clinical application and indication of CGM

As a new blood glucose monitoring technology, CGM examination is still relatively expensive, so in the clinical application process, it is necessary to master the indications and timing of monitoring, and make full use of its advantages, so as to maximize its clinical value.

The main advantage of retrospective CGM is the detection of occult hyperglycemia and hypoglycaemia not easily detected by traditional monitoring methods, especially postprandial hyperglycemia and nocturnal asymptomatic hypoglycemia. For example, (1) find blood glucose changes related to the following factors, such as food type, exercise type, drug type, mental factors, and lifestyle; (2) understand postprandial hyperglycemia, nighttime hypoglycemia, dawn phenomenon, and Somogyi phenomenon, which are difficult to find by traditional blood glucose monitoring methods; (3) help formulate individualized treatment plans; (4) improve treatment compliance; (5) provide a visual means for diabetes education. CGM has unique advantages in assessing blood glucose fluctuations and finding hypoglycemia.

Therefore, retrospective CGM is mainly suitable for the following patients or conditions, including: (1) type 1 diabetes mellitus and (2) the need for intensive insulin therapy (E. g., patients with type 2 diabetes treated by subcutaneous insulin injections or intensive insulin pump therapy); (3) patients with type 2 diabetes treated with hypoglycemic therapy under the guidance of SMBG, One of the following conditions remains: ① unexplained severe hypoglycemia or hypoglycemia with repeated episodes, Asymptomatic hypoglycemia, nocturnal hypoglycemia, ② Unexplained hyperglycemia, In particular, the fasting hyperglycemia, ③ Large blood glucose, ④ Out of the fear of hypoglycemia, Patients who deliberately maintain hyperglycemia; (4) gestational diabetes mellitus or diabetes mellitus combined with pregnancy; (5) Patient education: CGM, which can help patients understand the blood glucose changes caused by exercise, diet, stress, hypoglycemic therapy, etc., And thus can prompt patients to choose a healthy lifestyle, Improving patient compliance, Promote more effective communication between doctors and patients. Furthermore, diabetic patients with gastroparesis were treated with

And patients with special types of diabetes can also undergo CGM, if necessary, to understand the characteristics and changes of their blood glucose spectrum. Other endocrine and metabolic diseases with glycemic changes, such as insulinoma, can also apply CGM to understand the characteristics of glycemic changes. Type 1 diabetes, type 2 diabetes with intensive insulin therapy, and patients with large blood glucose fluctuations are the first population recommended for CGM. When appropriate, CGM can also be used as a valuable method for clinical research and outcome evaluation.

The principle of blood glucose monitoring of real-time CGM is similar to that of retrospective CGM. The main feature is to provide high and hypoglycemia alarm and warning functions while providing instant blood glucose information, so as to assist patients in real-time blood glucose regulation. The clinical positioning of the real-time CGM technique differ from the retrospective CGM technique. In 2011, the American Endocrine Society in conjunction with the Diabetes Technology Association and the European Endocrinology Society formulated the Guidelines for the Clinical Application of Dynamic Glucose Monitoring, which proposed the indication for real-time CGM. With the gradual and wide application of real-time CGM technology, the relevant clinical research results at home and abroad are constantly carried out, and the suitable population for real-time CGM is also under in-depth discussion. The current recommended indications are: (1) HbA 1c <7% of children and adolescents with type 1 diabetes, Using real-time CGM to assist with consistent patient HbA 1c levels, And does not increase the risk of hypoglycemia; (2) HbA 1c> 7% in children and adolescents with type 1 diabetes, If able to use and operate the instrument daily; (3) Adults with type 1 diabetes who have the ability to approach daily use; (4) type 2 diabetes patients hospitalized with insulin, Using a real-time CGM can reduce blood glucose fluctuations, Make blood sugar faster and more stable, It also does not increase the risk of hypoglycemia; (5) Patients with perioperative type 2 diabetes mellitus, Using real-time CGM can help patients better control their blood sugar; (6) Patients treated with insulin in non-intensive care units, Application of real-time CGM facilitates glycemic control and reduces the occurrence of hypoglycemia.

Specification for the use of the CGM

1. Accuracy evaluation

Because the CGM technique determines the glucose concentration of the subcutaneous intertissue fluid, but not the venous blood or capillary blood glucose values. Therefore, before the CGM data analysis after the monitoring, an important step is to first judge the accuracy of the monitoring results, only if the monitoring data is confirmed to be effective

Can be used to guide treatment options. The retrospective dynamic blood glucose monitoring system was included The "best accuracy" evaluation criteria of (CGMS) are: (1) 3 daily matched probe measurements and fingertip glucose values; (2) the correlation coefficient of daily matched probe measurements and fingertip glucose values is 0.79; (3) The difference between the maximum and minimum glucose is 5 mmol / L is 28%; when the difference between the maximum and minimum glucose is <5.6mmol / L, MAD is 18%..6

2. Normal reference value of dynamic blood glucose

At present, there are many relevant indicators of dynamic blood glucose, but no matter what the indicators, the principle is the statistical conversion and calculation of blood glucose value. The main difference lies in the different focus reflecting blood glucose level, blood glucose fluctuation and risk of hypoglycemia. Clinical applications should be specifically selected according to different evaluation purposes. There is a lack of internationally recognized standard for the normal value of dynamic blood glucose. The more reliable range of dynamic normal glucose values should be determined based on long-term prospective follow-up results as well as large natural population surveys. Before obtaining the above study results, the normal reference value of dynamic blood glucose can be tentatively determined based on the monitoring results of the normal population. According to the results of a national multi-center study conducted in China, it is recommended that 24 .6.8.9.9.4The mean h blood glucose value was <6 mmol / L, while the time percentage of 7 mmol / L and 3 mmol / L in 24h was <17% (4h) and 12% (3h), respectively; the mean blood glucose fluctuation amplitude (MAGE) and blood glucose standard deviation (SDBG) were <3 mmol / L and 1 mmol / L were used as the reference values for Chinese people. Meanwhile, the preliminary analysis showed that the 24h mean glucose value correlated well with HbA 1c, and when HbA 1c was 6.0%, 6.5% and 7.0%, the corresponding 24h mean glucose values of CGM were 6.6,7.2 and 7.8mmol / L, respectively.

3. Read the dynamic blood glucose map and the points of attention of the data

(1) When interpreting the results, we should focus on the analysis of the fluctuation pattern and trend of blood glucose, and try to find the possible causes of the abnormal fluctuation of blood glucose, rather than "tangled" the absolute blood glucose value at individual time points.(2) The monitoring data of each time only reflect the previous blood glucose control for a short time (such as 72h), and this time window cannot be expanded.(3) It is recommended to use the "three-step method" standard analysis mode to interpret the dynamic blood glucose map and data. Generally speaking, the first step is to analyze the nighttime blood glucose, the second step is to see the blood glucose before meals, and the third step is to observe the postprandial blood glucose. Each step is to observe hypoglycemia and hyperglycemia, and find the specific reasons to guide the adjustment of the treatment plan.

Appendix Specific glucose monitoring protocol examples

● Glucose monitoring protocol for patients treated with insulin

At present, most guidelines recommend that insulin-treated patients need blood glucose monitoring at least three times a day, and individual monitoring programs can be developed according to different treatments.

Table 1 Examples of blood glucose monitoring programs for patients treated with multiple insulin injections

blood	empty	After	ante	After	Before	After	at
XXXXX							
Has reached the standard	X				X	X	--

Note: X, the time to measure blood glucose;, can save the time to measure blood glucose.

A blood glucose monitoring regimen for intensive treatment of patients

Patients with intensive insulin therapy (multiple insulin injections or insulin pump) should monitor their blood glucose 5 to 7 times a day at the beginning of treatment, which is recommended to cover fasting, before and after meals, and before bedtime. If there is hypoglycemia, the blood sugar should be measured at any time. If unexplained fasting hyperglycemia or nighttime hypoglycemia occurs, monitor blood glucose from 2 to 3 points at night. After achieving the treatment target, blood glucose was monitored 2~4 times a day, mainly covering fasting and bedtime blood glucose, and after meals when necessary (Table 1).

Blood glucose monitoring protocol for patients treated with basal insulin

Patients using basal insulin should monitor fasting blood glucose every week 3d before reaching the blood glucose standard, once every 2 weeks, and test blood glucose at 5 time points 1d before the visit; monitor blood glucose 3 times a week after meeting the standard, namely fasting, breakfast and dinner, and test blood glucose spectrum at 5 time points 1d before the visit (Table 2).

Table 2 Blood glucose monitoring protocol for patients treated with basal insulin

blood sugar [illegible]	empty [illegible]	After [illegible]	ante [illegible]	After [illegible]	Before [illegible]	After [illegible]	at [illegible]
Not up to standard							
3d per week	X						
1d before the return visit	X	X		X		X	X
Has reached the standard							
3d per week	X	X				X	
1d before the return visit	X	X		X		X	

Note: X, the time required to measure the blood glucose.

Table 3 Examples of blood glucose monitoring protocol for patients receiving premixed insulin injections twice daily

blood sugar [illegible]	empty [illegible]	After [illegible]	ante [illegible]	After [illegible]	Before [illegible]	After [illegible]	at [illegible]
Not up to standard			per week		1 d b e f	ore the return visit	X

X

			X			
	X	X		X	X	
Has reached the standard						
3d per	X				X	X
week	X	X	X			X

Note: X, the time required to measure the blood glucose.

Glucose monitoring protocol for patients treated with premixed insulin twice daily

Patients with premixed insulin should monitor fasting blood glucose 3d before blood glucose every week, once every 2 weeks. It is recommended to take blood glucose profiles at 5 time points 1d before consultation, and monitor blood glucose 3 times a week, namely, fasting, before and after dinner (Table 3).

● Blood glucose monitoring protocol for patients treated without insulin

For non-insulin-treated type 2 diabetes patients, blood glucose monitoring frequency and regimen should be determined according to the treatment regimen and blood glucose control level. Generally, it can be monitored for 3d per week, and short-term intensive monitoring can be conducted in special circumstances.

Short-term intensive surveillance protocol for non-insulin-treated patients

Short-term intensive blood glucose monitoring is suitable for frequent hypoglycemia symptoms; stress status and treatment plan adjustment. The monitoring protocol was 3d per week to monitoring blood glucose at 5 to 7 time points daily, including before, after and bedtime (Table 4). With adequate glucose data and appropriate therapeutic measures, it can be reduced to an alternate glucose monitoring protocol (Table 5).

Meal-paired regimen for non-insulin-treated patients

The meal protocol is recommended to monitor blood glucose levels before and after breakfast, lunch and dinner (Table 6) to help patients understand the effects of diet and related therapeutic measures on blood glucose levels.

Table 4 Short-term intensive glucose monitoring protocol for non-insulin treated patients

time	empty stomac	After breakfast	ante prandium	After lunch	Before dinner	After dinner	at bed
Monday							
Tuesday							
Wednesday	X	X	√	X	X	X	√
Thursday	X	X	√	X	X	X	
Fri	X	X	√	X	X	X	√

Sat	
weekday	

Note: X, the time to measure blood glucose;, can save the time to measure blood glucose.

Table 5 Alternate self-blood glucose monitoring protocol for non-insulin treated patients

time	empty stomach	After breakfast	ante prandium	After lunch	Before dinner	Before going to bed after dinner
Monday	X	X				
Tuesday			X	X		
Wednesday					X	X
Thursday	X	X				
Fri			X	X		
Sat						
weekday	X	X			X	X

pour:X Time required for blood glucose measurement.
,

Table 6 Meal-paired blood glucose monitoring protocol for non-insulin treated patients

time	empty stomach	After breakfast, before lunch after lunch before dinner, after dinner before bedtime				
Monday	X	X				
Tuesday						
Wednesday			X	X		
Thursday						
Fri						
Sat					X	X
weekday						

Note: X, the time required to measure the blood glucose.
