# Supplementary material for: Efficacy of a smartphone application for helping individuals with type 2 diabetes mellitus manage their blood glucose: a protocol for factorial design trial
Source: Trials. 2023 Jul 22;24:468. doi: 10.1186/s13063-023-07489-5 (PMC10362696; doi:10.1186/s13063-023-07489-5)
Supplement: Supplementary file 3 — Additional file 3. [file 13063_2023_7489_MOESM3_ESM.pdf]

• clinical research •

# Correlation study between the level of self-management and quality of life in diabetic patients

Chen Ailing Zhang Zhenlu Liao Zhihong Wan Lihong Deng Wanping Yuan Yonghong

[Abstract] Objective To investigate the correlation of self-management level and quality of life in diabetes patients. Methods We used a descriptive correlation study with 214 patients using the Michigan Diabetes Management Rating Scale (DCP), the Michigan Diabetes Knowledge Test Questionnaire (DKT) and the Quality of life specific scale of Chinese diabetes patients (DSQL). Results (1) Self-management of diabetic patients includes: self-management ability, medication problems, exercise problems, attitude to disease, self-management persistence and I recognize management importance; dietary compliance, blood sugar or urine testing problems, condition control problems, understanding of diabetes knowledge. (3) In addition to blood glucose or urine glucose detection factors, the factors of self-management level were correlated with the DSQL score of survival quality 3 ($r = 0.174 \sim 0.641$, $P < 0.05$), and the higher the level of self-management of the above factors, the higher the level of their quality of life. Conclusion The level of positive positive associated with survival was wall.

[Key words] Diabetes; self-management; quality of life

*Self-management and quality of life in patients with diabetes mellitus CHEN Aiding .ZHANG Zhen-lu,UAO Zhi-hong,el al.The First Hospital Affiliated to Sun Yat-Sen University, Guangzhou 510080 ,China*

[Abstract] Objective To examine the relationship between self-management and quality of life in diabet ics.Methods A descriptive and correlation design was used in this study.214 subjects completed the Universily of Michigan Diabetes Research and Training Cenler Diabetes Care Profile ( DCP), the Diabetes Knowledge Test (DKT), and the Diabetes Patients' Quality of Life in China (DSQL).Results ( 1) The diabetics reported higher levels of self-management for 6 of 11 domains.They were self-management ability, medical barriers, exer cise barriers.attitude towards diabetes, self-management adherence, and importance of self-management.Howev er, they reported lower 1 evels of self-management for other 5 domains.They were diet adherence.monitoring barri ers, control problems.understanding diabetes knowledge, and DKT scores.(2) The mean score of quality of life by DSQL was 54.75 x 13.05. (3) The absolute value of correlation coefficients between 10 domains' mean scores of self-management (except of monitoring barriers) and DSQL total score ranged from 0. 174 to 0.641. There was a positive correlation between self-management and quality of life in patients with diabetes.Conclusion Overall, most diabetics reported a moderate to low level of self-management and quality of life.There was a moderate and positive correlation between self-management and quality of life.

[Key words] Diabetes mellitus ; Self-management; Quality of life

According to the International Diabetes Federation (IDF) estimated that (1), there are about 40 million diabetic patients in China, higher than that in western developed countries. Domestic and foreign studies have shown that'23], the level of self-management is positively correlated with glycemic control, and a good self-management level can reduce and delay the occurrence and development of complications. The so-called self-management is to take the initiative to adjust their psychological activities and behaviors, control improper impulse, overcome the adverse situation, seek development, and achieve good adaptive quality, its quality, that is, the level of self-management (4). Studies have found that in (1), a good level of self-management is important for maintaining a relatively normal quality of life. In this study, descriptive correlation study was used to explore the self-management level and quality of life of diabetic patients, and then clarify the relationship between the two, so as to provide a strong basis for the intervention methods to improve their self-management level and even the quality of life.

Fund Project: 2002 Central Health Care Commission (B039) Author: 510080 Guangzhou, Department of Endocrinology, the First Affiliated Hospital of Sun Yat-sen University (Chen Ailing, Jian Zhihong, Deng Wanping, Yuan Yonghong), Nursing Department (Zhang Zhonglu); School of Nursing, Sun Yat-sen University (Wan Erhong)

## Object and method

one, target

In this study, 214 Chinese patients with diabetes mellitus met the latest diabetes diagnosis and classification criteria revised by the American Diabetes Association (ADA) in 1997. The subjects were obtained from patients with type 1 and type 2 diabetes who were hospitalized in the endocrine department and

specialist outpatient clinics of the First Affiliated Hospital of Sun Yat-sen University between October 2003 and August 2004. Excluded: ① confusion, uncooperative, unclear language; ② with other serious heart, brain, and lung diseases; ③ with neurological disorders or psychiatric history. General situation of subjects: type 1, 8.0%, 92.0%; 108 outpatients, 106 inpatients; female 55.60%, male 44.4%; most concentrated in high school, 65.40%; more married, 94.8%; age 19-74 years; disease course of 0.1 ~43 years.

two, method

In this study, interview and questionnaire survey are used to collect data, the author in the outpatient opening time or the free time of ward patients to the questionnaire, let the patients to fill in, 30-40min; for reading difficulties or visual impairment, the author does not add any prompts to read, let the patients choose, record one by one, 50 ~60

1. Measurement tool: Landscape survey tool includes the following aspects: ① Evaluation scale of self-management level'6 (7): Self-management level of diabetes patients was determined using the six subscales of Michigan Diabetes Management Rating Scale (DCP) and the Diabetes Knowledge Test (DKT Questionnaire (DM), Thus the level of self-management in this study includes self-management behaviors, Knowledge knowledge of diabetes mellitus, And the attitude, adherence and awareness of the self-management of diseases, There are 11 factors: 1 self-management ability; 2. Diet compliance; 3. Drug treatment problems; 4. Exercise problems; 5. Blood sugar or urine sugar detection problems; 6. Disease control problems; 7. Self-assessment of the understanding of diabetes knowledge; 8. Diabetes knowledge test (DKT) score; 9. Attitudes towards illness; 10. Self-management persistence; 11. Understanding of the importance of self-management. DCP and DKT are maturation scales for the development of the Diabetes Research and Training Center (MDRIC) in Michigan, USA, and the Cronbach a coefficient of DCP measured by Fitzgerald et al is above 0. 60 and up to 0. 95。The Cronbach a coefficient per DKT question is above 0.709E. DCP and DKT are used in Chinese, edited by Liao Zhihong, a clinical expert in diabetes research. ② Quality of life specific scale (DSQL)[183]: DSQL mainly reflects the impact of diabetes and its treatment on patients' physical, psychological and social relations, four dimensions: the impact of physiological function, psychological, mental, social relations and treatment g DSQL total score 120, the lower the score, the less affected by the disease, and the better the quality of life. DSQL, with good reliability, validity and responsiveness, where the Cronbach a coefficient is 0.945 and the half reliability is 0.91。The Cronbach a coefficient of the pre-test was 0.95.

2. Data analysis: All data were analyzed in SPSS11. Software processing. The self-management level and quality of life were described by mean and standard deviation, and the correlation analysis of self-management level and quality of life adopts Pearson correlation analysis.

bear fruit

one, The level of self-management in patients with diabetes mellitus

In this study, a total of 11 factors evaluated self-management in their self-management behavior, mastery of diabetes knowledge, attitude towards disease self-management, and adherence to self-management. The mean scores of the 11 factors are shown in Table 1. The results showed that the level of self-management of diabetic patients was not ideal, and the level of different factors varied C

two, Quality of life of patients with diabetes

The DSQL score for QoL of diabetic patients was (54.75 ± 13.05), with 3.7% of patients with lower level (N80 points), 83.7% with moderate level (40 w DSQL <80) and 12.6% with higher level (<40 points)。See Table 2.

three, Correlation analysis of the level of self-management and quality of life in diabetic patients

With the 11 factor scores of self-management level and the DSQL score of quality of survival to do pearson, correlation analysis, found DSQL, score divided with

*Table 1 DCP, although Table and DKT scale scores (score, x ± s, n = 2l4)*

| Dimension / due to | *its* |
|---|---|
| Self-management behavior | |
| Self-management ability | 3.55 ±0.95 |
| Diet compliance | 2.04±1.61 |
| Drug treatment problems | 1.37 ±0.59 |
| Exercise | 1.76 ±0.63 |
| Blood glucose or urine sugar [illegible] | 1.63 ±0.53 |
| Disease control disease [illegible] | 2.6611.1! |
| Knowledge of fine disease | |
| Self-assessment of the [illegible] | 1.95 ±1.43 |
| DKT score | 57.54 ±26.04" |
| Attitude, persistence, and [illegible] | |

| | |
|---|---|
| Attitudes towards disease | 3.01 ±0.64 |
| Self-management and | 3.28 ±0.85 |
| Understanding of the | 4.33 ±0.65 |

Note: P <0.05

Table 2 DSQL score of quality of life of diabetic patients (score, x ± s, n = 214)

| dimension | |
|---|---|
| DSQL grade | 54.75 ±13.05 |
| Physiological dimension | 20.83 ±5.71 |
| Psychological / spiritual | 20.42 ±6.28 |
| Social dimension | 7.12 ±2.68 |
| Therapeutic dimension | 6.39 x 1.70 |

There was no significant difference in the correlation score of blood glucose or urinary glucose detection factors, but the correlation with other factors was statistically significant, indicating that the higher the level of self-management, the higher the quality of life of the patients. See Table 3.

Table 3 Correlation between the factors of self-management level and quality of life (mine)

| Self-management level factor | DSQL grade |
|---|---|
| Self-management ability | -0.448* * |
| Diet compliance | -0.236 •- |
| Drug treatment problems | 0.390* , |
| Exercise exercises | 0.339 • . |
| Blood sugar or urine prevarication | 0.061 |
| Disease control problem | 0.251** |
| Self-evaluation of the | -0.174* |
| DKT score | -0.214* * |
| Attitudes towards disease | -0.641 •• |
| Self-management and persistence | -0.419* * |
| Self-management car to understand | -0.253*" |

Note: P <0.05, * * P <0.01

send army or despatch troops to suppress or assault The Analects of Confucius

As the results from the results, Patient self-management ability in this study was at a moderate and high level, This is consistent with foreign reports of similar studies such as Fitzgerald and (6), But that patients perform specific self-management behaviors, Such as diet, exercise, drugs, monitoring, etc., Different levels of overcoming problems: diabetic patients have fewer problems in medication or exercise; yet, Their poor dietary compliance, Meanwhile, the actual scores of 214 patients were statistically treated, Found that 168 cases (78.5%) without testing for blood, urine sugar, 117 (54.7%) had no monitored disease, Leading to a blind 0 treatment, Can effectively control blood sugar. In terms of diabetes knowledge, the score of DKT was at low and low level, which is similar to that reported by (7). It shows that there are still more patients who lack knowledge or cognition of diabetes. In addition, the patients in this study had a positive attitude towards disease manifestations, and the results of this study were similar to those reported by (6). At the same time, more patients are aware of the importance of long-term control of blood sugar, body quality and bad mood. At present, scholars at home and abroad have extensively studied the survival quality of diabetic patients, and the results report that the quality of life of diabetic population is generally low and lower than that of normal population[9,12]0 And although the measurement tools of quality of life are different, they all show that the quality of life of diabetic patients is significantly reduced. In this study, DSQL was used to adjust diabetic patients and found that their quality of life was at a low level, and this result is similar to that reported by Zhou Ming et al. This study found that the level of psychological or mental, physiological and social functions were low, so their quality of life needs to be improved and improved. The important purpose of this study is to clarify the relationship between the self-management level and the quality of life of diabetic patients. The pearson correlation analysis showed that the 10 factors of self-management level were closely related to the quality of life. Self-management ability, self-management ability, dietary compliance, understanding of diabetes knowledge, DKT, attitude towards disease, characteristics and importance of self-management, the higher the score of the quality of life; similarly, the lower the score of drug treatment, exercise and condition control problems, the higher the quality of life. All this indicate that there is a positive relationship between the level of self-management of diabetes and quality of life of patients, which is consistent with the report of Parchman et al., that is, patients with higher level of self-management have higher quality of survival and level.

The results of this study found that the patients' attitude towards disease score and DSQL score correlation coefficient of absolute debt, 0. 641, shows that there is no positive and optimistic attitude, patients can not actively participate in diabetes education, can not master diabetes treatment self management knowledge and skills, more not lifelong treatment, the quality of life will decrease, further hinder the treatment of the disease, thus forming a vicious circle. This suggests that in the

future diabetes education, attention should be paid to the intervention of patients' cognitive behavior methods, so that patients can cultivate a positive, optimistic and brave attitude, and improve the quality of life.

The results of this study also showed that self-management ability score and self-management adherence score showed a moderate negative correlation with DSQL score, indicating that maintaining good self-management ability and long-term adherence to treatment can reduce the damage caused by the disease, maintain normal life, and then improve the quality of survival. The high level of self-management ability and self-management adherence of patients in this group suggests that specialized workers should continue to strengthen the self-management ability of guiding patients, strengthen and change the significance of long-term self-management, so as to maintain the relatively normal survival site of patients. This study found that the drug treatment problem score of diabetic patients and exercise problem score were moderately positively correlated with the DSQL score, indicating that long-term adherence to drug therapy, maintain good and medical adherence behavior, and adhere to reasonable exercise is of great significance to improve the quality of life.

The results showed that the absolute value of the correlation coefficient between DSQL score and dietary compliance score, score of condition control problem score, DKT score, and score of recognition of the importance of self-management were small, between 0.2 and 0.3. Through the analysis, the above factors generally with DSQL social relationship dimension score, the dimension of correlation no statistical difference c, therefore, the patients demanding high disease treatment, although in diet control, condition monitoring, knowledge and cognitive management level is high, but failed to improve its quality of life in social and treatment. This suggests that in the diabetes education should grasp the scale, guide the correct understanding of the disease, the use of diabetes knowledge, self-management, to avoid misunderstanding caused by a little knowledge, and excessive control of diet and worry about the disease and cause adverse effects on the physical and psychological patients. The results of this study found that the correlation coefficient of DSQL score and understanding self-assessment score of diabetes knowledge was-0.174, which may be related to the degree of patients' own requirements and subjective awareness. Therefore, there exists and the influence of confounding factors such as subjective awareness, and the relationship needs to be further explored.

Previous studies have found that the self detection of blood, urine sugar patients the quality of life is higher, these patients can timely understand their own blood sugar, effective prevention and delay the occurrence of complications, so as to improve the quality of life, however, this study found that the correlation between the two statistical significance, is likely to be 78.5% of these patients without self detection of blood, urine sugar. The relationship between the two needs to be further explored.

## reference documentation


I Mokdad All , Engelgau MM, Ford ES.Diabetes trends in the US.: 1990-1998. Diabetes Care, 2000, 23： 1278-1283.

2 years, Yin Zhimiao, Yao Dongfang, et al. Adjustment analysis of self-management ability of patients with speruria. Journal of Nursing, 2001,16:648-649.

3 Jones H, Rossi JS, Edwards L, el al.Changes in diabetes self-care be haviors make a difference in glycemic control : the diabetes stages of change （ DiSC ） study.Diabetes Care.2003,26： 732-737.

4. Wang Yiming, Jin Yu. Review of the self-management research.psychic science.2002,25=453456.

5 Glasgow RE, Toobert DJ.Brief, computer-assisted Diabetes dieiary self management :counseling effects on behavior, physiologic outcomes, and quality of Life.Medical Care, 2000 , 38： 1062-1073.

6 Fitzgerald JT, Davis WK, Connell CM, et al.Development and valida tion of lhe Diabetes Care Profile.Evaluation and the Health Plofessions, 1996.19:209-231.

7 Fitzgerald JT, Anderson HM, Funnell MM.ct al.The reliability and validity of a brief Diabetes Knowledge Test.Diabetes Care, 1998 , 21: 706-710.

8 Zhou Fengqiong. Diabetic quality of survival, specific scale. See: Editorial Board of Behavioral Medicine Sciences, China. A parallel manual of behavioral medicine. Beijing: China Medical Electronic Audio-Video Publishing House, 2005.114-116.

9 Koopmanschap M.Ceding with Type 2 Diabetes : the patient' s perspec tive.Diabetologia, 2002 , 45： 18-22.

10 Zhou Ming. Chen Wei. Lin redundancy, etc. The survival quality of patients with buuria disease in Meizhou city. Clinical Rehabilitation in China, 2002,6:1614-1615.

11 Pan Runde, Zou Tao. Study on the correlation of mental health status and influencing factors in patients with type 2 diabetes '. Chinese Behavioral Medicine Sciences, 2004,!3：36.

12 Sun Bing, Sun Hailing, Banbo, et al. Effect of behavioral intervention on control of quality of life, condition and metabolic index in type 2 diabetes. Behavioral Medicine Science in China, 14:175177..2005
