# Supplementary material for: Efficacy of a smartphone application for helping individuals with type 2 diabetes mellitus manage their blood glucose: a protocol for factorial design trial
Source: Trials. 2023 Jul 22;24:468. doi: 10.1186/s13063-023-07489-5 (PMC10362696; doi:10.1186/s13063-023-07489-5)
Supplement: Supplementary file 4 — Additional file 4. [file 13063_2023_7489_MOESM4_ESM.pdf]

Central South University

Master's L degree thesis

Compliance and its influencing factors in rehospitalized diabetic patients

His surname is Chen Yamei

Application for Degree level: Master's degree I:

Major: Nursing

Advisor: Gold

20070101

Objective: L To assess the compliance status of patients rehospitalized with diabetes (diabetes mellitus, DM), and found the overall characteristics and general rules of compliance. 2 . To the shadow of compliance in rehospitalized DM

• ▪

Sound factor, the analysis of the influence of each factor on its compliance.

Methods: This study is a case-control study, using a random sampling method from April 2006 to 2

In September 2006, 107 cases of primary hospitalized DM patients and 194 re-hospitalized DM patients were administered in seven comprehensive hospitals in Changsha, Hunan Province. The survey tool includes five questionnaires: the general data questionnaire to collect the basic information of patients; the treatment compliance questionnaire evaluates patients' treatment compliance; the DM knowledge questionnaire, the health belief questionnaire and the social support rating scale to measure patients' knowledge level, health belief and social support, and the results of first-hospitalized DM patients (first-hospitalized group) and re-hospitalized DM patients (re-hospitalized group). Using SPSS 11.5 statistical software, descriptive analysis, analysis of variance, t-test, I? Testing, Pearson correlation and analysis, and unconditional Logistic regression analysis.

Results: 1. The results of the two groups' t-test showed that the total

compliance score of primary and re-hospitalized DM patients were statistically significant, exercise and self-monitoring scores (p <0.05) o Both groups had the highest score in drug treatment dimension and the lowest score in regular review dimension..

2. The results of the two groups showed that the difference in the knowledge level between the initial and re-hospitalized DM patients was statistically significant (p <0. 001) o

3. Did the two groups of hospitalized DM patients participate in DM education evaluation and pass Jr? Test, the results showed a statistically significant difference between primary and rehospitalized DM patients receiving DM education (p.001) o

4. The results showed that the most influencing factors were: health belief, social support, education level, age, and menstruationEconomic status, DM knowledge, and their regression coefficients were 1.145,0.641, -0.550,0. 541,0.493,0. 465, respectively, with the most influential factor was health belief.

Conclusion: 1. DM patients re-hospitalized had better compliance than first-hospitalized DM patients, and the total scores, exercise and self-monitoring scores of compliance assessment were all higher than those of first-time hospitalization. Among the five dimensions of treatment compliance among rehospitalized DM patients, followed by diet, exercise, self-monitoring, and regular review of compliance.

2. The knowledge level of re-hospitalized DM patients was higher than the first-hospitalized DM patients.

3. Rehospitalized DM patients receive DM health education status is better than primary hospitalized DM patients.

4. The main factors affecting the treatment compliance of rehospitalized DM patients were: health belief, social support, education level, age, economic status, and DM knowledge.

Key words: diabetes, compliance, influencing factors, health education

Chapter II: Research Objects and Methods

2.1 Study subjects

This study selected from April 2006 to September 2006 in Changsha city of Hunan province seven comprehensive tertiary hospital, including Xiangya a, two, three hospitals, Hunan province people's hospital, Changsha central hospital, Changsha city first, three people's hospital of endocrine specialist hospitalized DM patients, a total of 301 cases, including 107 cases of the first hospitalized patients, 194 hospitalized patients again. Primary inpatients are patients who have confirmed DM for at least half a year and are hospitalized for the first time with DM. Repeat patients were patients with DM and a second or more hospitalization for DM. Inclusion criteria: (1) meet the diagnostic criteria for DM established in the WHO1985 year. (2) DM for at least half a year. (3) No cognitive or mental disorders. (4) Have a certain reading ability. (5) Willing to participate in the adjustment.

2. 2 Study Methods

2.2.1 Study design

This study was designed as a questionnaire questionnaire study using random sampling to adjust treatment compliance and influencing factors in primary and re-hospitalized DM patients.

2.2.2 Measurement tools

The questionnaire used in this study included five parts, namely, general data scale, treatment compliance questionnaire, DM knowledge questionnaire, health belief questionnaire and social support rating scale.

2. 2.2.1 General data adjustment table

General data adjustment table is designed by the investigator, including gender, age, marital status, occupation, education level, economic income, medical expenses, family medical expenses, DM type, disease course, health education, fasting blood glucose (FBS), 2 hours postprandial blood glucose (2 hBS), glycosylated hemoglobin (HbAC), etc. Among which, FBS, 2 hBS, HbA. C The case was reviewed by the investigator, and the first test index was recorded

on admission.

2.2. 2.2 Treatment compliance Questionnaire

In this study, referring to Li Xueqin's DM compliance questionnaire, we repeatedly consulted expert modification to revised the compliance questionnaire from five aspects: medication, diet, exercise, self-monitoring, and regular rehabilitation. The questionnaire included 20 items and were evaluated from the above five dimensions. Medication adherence includes four questions: ① Have you ever forgotten to take your medication or inject insulin?② Have you ever heard of the persuasion of non-medical staff to change the medication by yourself?③ Have you ever reduced your dosage or stopped your medication when your symptoms improve or your blood glucose control is good?④ Have you ever increased your dosage or changed your medication when your symptoms are worse or your blood sugar value is high? Dietary compliance includes four questions: ① Do you eat irregularly?② Your diet type is single, can not be adjusted in time when the condition changes?③ Did you arbitrarily add food when your blood sugar control is good?④ Do you deliberately eat less or no food when your blood sugar control is poor? Exercise compliance includes four questions: ① Have you ever stopped exercising for various reasons?② Do you usually play mahjong, play cards, watch TV or sit still for a long time as the main way of leisure?③ Do your daily work or housework be your main exercise?④ Do you do outdoor activities for 3,060 minutes a day, or at least 2 hours a week?(Such as walking, playing tai Chi, etc.). Self-monitoring compliance includes four questions: ① Do you self-monitor blood sugar when climate change, poor diet, stress, mood swings and other changes?② Have you ever stopped monitoring your blood sugar for more than 1 month because of feeling good?③ You only measured blood sugar regularly, not blood pressure, blood lipids, HbA 1 c, etc.?④ Do you not adhere to blood sugar monitoring when going out or traveling? Regular compliance includes four questions: ① Have you ever forgotten to review?② You go to the hospital when your condition deteriorated.③ Do you think you can feel the level of blood sugar, but did not review as required?④ Have you not reviewed for more than 3 months due to various reasons?

Each item of the questionnaire was scored using the level 3 scoring method, namely, often (1 point), occasionally (2 points), and never (3 points). The highest theoretically possible score was 60 points, with a higher score indicating better patient compliance. The score of each dimension is equal to

the sum of the dimensional entry scores. In addition, motor compliance entries 12,13 used reverse scoring, i. e., often (3), occasionally (2) and never (1).

In this study, the criterion of treatment compliance degree: according to the average compliance score (40 points) as the cut-off point, the score of 4060 is good compliance, and <40 is poor compliance.

In this study, the content validity (content validity index, CVI) was assessed before the formal investigation. Five DM experts and nursing experts were invited to score and provide opinions on the applicability, clarity and coverage of each item from the four-point method of "no correlation" to "highly relevant", and the CVI value was 0.83. Meanwhile, two pretests were conducted in 24 DM patients, and the test-retest reliability of the questionnaire was 0.86 and Cronbach * sa was 0.86, where the Cronbach's a of questionnaire drug, diet, exercise, self-monitoring, and regular recompliance was 0.85,0.84.0.79,0.84,0.78, and 0.82, respectively, indicating that the questionnaire had good reliability and validity.

2.2.2.3 DM knowledge questionnaire

In this study, we modified (iv) by referring to the DM knowledge applied by the World Health Foundation China DM Education program. This questionnaire includes 25 items of DM, including symptoms, inducing factors, treatment, prevention, and self-care, etc. The scoring method is 1 point for each correct answer, and the total score is accumulated. This questionnaire CVI value of 0.80 and test-retest reliability of 0.75 were within the acceptable range of the study.

2.2.2.4 Social support Rating scale

The social support rating scale compiled by (iv) was used in this study. Social support refers to the spiritual and material help and support from all aspects of society, including family, friends and others, when a person is ill. The scale consists of 10 items, including three dimensions: subjective support, objective support, and social support

Use degree. The scale has fewer items, facilitates the subjects to answer, and has good reliability and validity.2.2.2.5 Health Beliefs Questionnaire

In this study, Rosenstock, Becker and others used the main arguments of the relevant literature as the theoretical framework of the health belief questionnaire to design the DM health belief questionnaire itself. Health belief

mode (health belief model, HBM) is an important theoretical mode to explain people's health behavior factors from the perspective of the formation of people's health belief. It was proposed by some American psychologists in the 1950s and developed after long-term research by social psychologists such as Becker, Rosenstock and Kegeles.

This questionnaire analyzed the relationship between the health beliefs of DM patients and their treatment compliance from five aspects: "the benefits of DM treatment, the harm of DM treatment, the necessity of DM treatment, the motivation of maintaining body health, and the harmfulness of DM". The questionnaire is designed with 20 items, using Likert^The 5-level scoring method, namely 1= very approve, 2 = approve, 3= uncertain, 42 approve, 5= very approve. Of these, 814 entries adopt reverse scores, namely 5= very disapprove, 4= disapprove, 3= uncertain, 2= approve, and 1 = very approve. The highest theoretically possible score is 100, and the higher the score, the stronger the patient's health beliefs. Each dimension is scored as the sum of the scores of each dimension item. The questionnaire invited five DM experts, psychology experts and nursing experts for the applicability, clarity and coverage of each topic, each topic from "complete, unrelated" to "high correlation" to score and provide opinions, measured the CVI value of 0.8L calculated the retest reliability of the questionnaire is 0.82, Cronbach'sa is 0.79, indicating that the reliability is within the acceptable range of the study.

2. 3 Data collection

Before the formal investigation, the 24 cases of DM patients were investigated, and then the survey has the first and fill again DM patients, fill in the questionnaire time for the patients in the hospital, all the researchers to meet the inclusion criteria, to explain the purpose and significance of the survey, obtain the trust, support and consent after the questionnaire, the patients are completed within 1 hour after the questionnaire, and immediately after the grave recovery. I give appropriate explanation to the items that have difficulty in understanding, and keep the explanation content of each item-to. For older patients with poor vision, the researcher will read to the patients one by one, and then the patient himself will make a choice, and the researcher will not give any induction.

2.4 Statistical treatment

Use SPSS11.5 statistical software to establish a database, data entry, sorting, statistical processing and analysis. Statistical methods mainly include: descriptive analysis, two-sample t check Jun. One-way ANOVA, 7 tests, Pearson correlation analysis and non-conditional Logistic regression analysis. Test level a =0.05, P value of all

The ratio of the two sides of the virus was taken[1, 3J]0

2. 5 Quality control

1. Quality control of the study questionnaires.

On the one hand, authoritative questionnaires are widely used at home and abroad, such as social support scale. On the other hand, referring to the domestic and foreign influential and representative questionnaire, combined with the research objects and objectives, such as the compliance questionnaire. On the other hand, researchers applied the widely recognized famous theoretical model as a theoretical framework to read a large number of literature and consult experts, and designed questionnaires, such as health belief questionnaire. In addition, before the official transfer, I made pre-adjustment in the endocrine ward of the second Xiangya Hospital, and made full use of the feedback information of the pre-survey to modify and improve the questionnaire, consulted experts again, and finally determined as the official questionnaire.

2. Data acquisition and quality control.

(1) All questionnaires were distributed and collected by the researcher. The researchers held a scientific and rigorous working attitude and adopted the procedures of unified distribution, interpretation, explanation and questionnaire collection to eliminate the variation among different observers.

(2) After obtaining the consent and promising the confidentiality of all the respondents by the investigator, the questionnaire was given out at the bedside of the ward and collected on the spot. Keep the adjustment environment quiet, and the patient is emotionally stable.

(3) At the end of the adjustment, check the integrity of the questionnaire

immediately. If the wrong, missing or illogical items are found, re-ask to supplement or correct.

3. Data processing and analysis of QC.

Before the data analysis, the coding and input work of the data were wrong, missing and logical check. Adopt repeated input method, establish nuclear document program, reduce the human error of data input, and ensure the reliability of data.

Append ix 5 Part V: The Health Beliefs Questionnaire

This section concerns your views of health, expressing your degree of approval or disapproval of column F issues. Please select one of the five alternatives and type ″ · in the mouth

1. I think persistence in treatment can reduce serious complications such as kidney disease, cardiovascular disease and eye diseases.

Very much [illegible] Do not [illegible] Unsure [illegible] In favor [illegible] Very much [illegible]

2. If you see a doctor regularly I believe the doctor will help me control my blood sugar

Very much disapprove of it Do not agree with mouth Unsure In favor of mouth Very much in favour□

3. Early treatment of diabetes is much cheaper than the treatment of comorbidities

Very much disapprove of it Do not agree with indeterm [illegible] In favor of mouth Very much in favour

4. Good diet control and medication will better control the hyperglycemia

Very much disapprove of it disfavour □ Unsure In favor of mouth Very much in favour

5. Being able to discuss your illness with your doctor will be of great help to your health

Very much [illegible] Do not [illegible] Unsure [illegible] In favor [illegible] Very much [illegible]

6. I think taking hypoglycemic drugs or insulin injection can reduce or eliminate the physical symptoms caused by disease.

□ Yes very strongly yes□

7. Although diabetes is a chronic disease, such as long-term treatment adherence, blood sugar can be improved and controlled.

Very much disapprove mouth disapprove□Not sure in yes Very much in favour□

Very disapproving mouth disapproving mouth uncertain mouth disapproving mouth Very much in favour of the mouth

9. There are diabetes caused by complications, control or not control of blood sugar are the same.

Very disapproved□ Disapproving, inconclusive, unapproving mouth Very much in favour of the

10. Diabetes is a chronic disease, and it is troublesome to take medicine for a long time. In order to facilitate, take medicine only in the body.

Very disapproved□Unyes very yes

11. Since there is no drug to cure diabetes, it is useless even to take it for a long time.

Very much disapprove mouth disapprove□Unsure yes very yes

12. Since diabetes is high blood sugar, once blood sugar drops to normal, there is no

Very disapproved□ Disapproving, inconclusive, unapproving Very much in favour need to continue taking medication.

13. I am worried that long-term medication will have side effects on the body.

Very much disapprove mouth disapprove□ indeterminacy□In Very much in favour of O favor of mouth

14. I worry that long-term insulin injections will be Very much in favour□

The purpose of a doctor is to cure diseases. His advice is good for my

Very much disapprove of it Do not agree with the mouth Unsure yes very mouth

16. I think the medical staff guide more than newspapers Information provided by television, or other media, follow.

Very much disapprove mouth disapprove mouth Not sure in yes Very much in favour of the mouth

17. I should often monitor the blood ponds to understand or find out my health problems early.

Very much in favour of the mouth

Very disdisdisdisnot uncertain □ number into mouth

Very disapproved□ Disapproving, inconclusive, unapproving Very much in favour□

19. I am worried that diabetic people who do not adhere to the medication will cause blood sugar fluctuations, which will affect my daily life.

Very disapproved□Disapproving unsure yes very supportive

20. I am worried that a high blood sugar will affect my study and work.

Very disapproved□ Disapproving unsure yes very supportive
