# Supplementary material for: Efficacy of a smartphone application for helping individuals with type 2 diabetes mellitus manage their blood glucose: a protocol for factorial design trial
Source: Trials. 2023 Jul 22;24:468. doi: 10.1186/s13063-023-07489-5 (PMC10362696; doi:10.1186/s13063-023-07489-5)
Supplement: Supplementary file 5 — Additional file 5. [file 13063_2023_7489_MOESM5_ESM.pdf]

airiti Library

The DOI digit object ID is registered in this article * 5 The butterfly

▶ Discussion of self-care behavior and related factors of non-insulin-dependent diabetes patients in the initial stage of outpatient diagnosis

Self-Care Behaviors and Related Factors in Outpatients Newly Diagnosed with Non-Insulin-Dependent Diabetes Mellitus

doi:10.6224/JN .45.2.60

Journal of Nursing, 45 (2), 1998

The Journal of Nursing, 45(2), 1998

Author / Author: Jingxuan (J.S. Wang); Wang Ruixia (R.H. Wang): Lin Qiuju (C.C.Lin)

Pages / Page: 60-74

Publication date / Publication Date: 1998A) 4

When quoting this article, please provide DOI information and obtain the most correct bibliographic information through the DOI permanent website.

To cite this Article, please include the DOI name in your reference data.

Please use this document DOI permanent website to link:

To link to this Article:

http://dx.doi.org/10.6224/JN.45.2.60

DOI is the abbreviation of digital object identification code (Digital Ob^ct Identifier, DOI), which is the only identification code on the Internet in this article,
For permanent links and references to this article.

To learn more about DOI use,
Please refer to the http: / / doi.airiti.com

For more information,
please see: http://doi.airitj.com

VOI

Please scroll down to the next page- -start reading this article

PLEASE SCROLL DOWN FOR ARTICLE

The most complete, academic, integrated query platform one Huayi online library http: / / www.airitilibrary.com • Find more academic articles on Airiti Library

Non-insulin-dependent diabetes mellitus patients at the beginning of the outpatient diagnosis Study of self-care behavior and its related factors

Self-Care Behaviors and Related Factors in Outpatients Newly Diagnosed with Non-Insulin-Dependent Diabetes Mellitus

Wang Jingxuan, Wang Ruixia, Lin Qiuju

Ms.Wang Jingxuan: Master of the Institute of Nursing, Kaohsiung Medical Hospital, currently lecturer in the Department of Nursing, Kaohsiung Medical Hospital. Ms.Wang Ruixia: Master of Medical Research Institute, Kaohsiung Medical Hospital, currently associate professor in the Department of Nursing, Kaohsiung Medical Hospital. Ms.Lin Qiuju: Master's degree in Nursing at Winona University. She is currently a lecturer in the Department of Nursing of Kaohsiung Medical Hospital.

abstract

This study is a related study to understand the self-care behavior and related factors of non-insulin diabetic patients. From December 10,1985 to February 26,86, a structural questionnaire scale was collected for diabetes patients of a medical center in Kaohsiung, and a total of 130 valid questionnaires were obtained. The results found that: (1) among all self-care behaviors, the percentage of high and low self-care behaviors was the lowest, only 49.88%.(2) Social support and self-efficacy were both significantly and positively correlated with self-care behavior.(3) In the stepwise regression analysis, the best predictor of self-care behavior was self-efficacy, explaining 74.0% of the variation. Based on the results of this study, it can be used as a reference for future diabetes health education and provide suggestions on future research directions.

Key words: self-care behavior, self-efficacy, social support, non-insulin-dependent diabetes mellitus.

preface

With the change of population structure, drunk food and life patterns in Taiwan, diabetes has become one of the most chronic diseases in Chinese people. According to the study of Dai (Min 82), the prevalence of non-insulin-dependent diabetes in Taipei was 8.17% and 5.06% in Taipei, while the incidence of non-insulin-dependent diabetes over 40 was 15 per 1,000 people per year. Thus, the severity of diabetes threatening the health of Chinese people cannot be ignored.

The treatment plan for non-insulin-dependent diabetes is as complex as insulin-dependent diabetes, and patients need patient, long-term and continuous treatment. For diabetics, daily self-care is a special challenge because the complex and essential self-care behaviors can change their life patterns, including complex drunk food treatment, weight control, blood glucose monitoring, foot care, and oral hypoglycemic drugs or insulin injections (Polly, 1992). However, while home-based self-care is quite important for people with diabetes, but like other patients with chronic diseases, diabetes patients often fail to do it well effectively (Bloom Cerkoney, & Hart, 1980).

In diabetes patients WeiJiao research, confirm the factors affecting self care behavior is the main topic (Irvine, Saunders, Blank, & Carter, 1990) ° and contact with the patient and most time caregivers, if can in the sugar, urine disease disease early, understand what, factors can affect the patient home self care behavior and assist it, execution, can help patients to establish a good home, gu mode, and achieve good blood sugar control and prevent the occurrence of complications.

At present, non-insulin-dependent diabetes patients in Taiwan can be 98% -99% of all diabetes patients, and the proportion is higher with the annual growth, and the prevalence rate of the elderly over 70 years old is 12% (Dai, 82). Domestic and foreign literature on ■ discusses the factors affecting self-care behavior of diabetes patients, including adolescents with insulin-dependent diabetes (Peveler, Davies, Mayou, Fairburn, & Mann, 1993; Xiao et al., Min 83). However, due to ethnic groups, age and disease differences, the results obtained from adolescents or adults with insulin-dependent diabetes are not universally applied to non-insulin-dependent diabetes patients (Wilson et al 1986). Although related factors affecting self-care behaviors vary with the period of illness (Connelly, 1987); however, in many studies, the average duration of diabetes is more than five years (Wilson et al., 1986; Glasgow, & Toobert, 1988; Zhan, Min 72), even more than ten years (Wooldridge, Wallston, Graber, Brown, & Davidson, 1992; Polly, 1992; Irvine, Saunders, Blank, & Carter, 1990), so the results are difficult to apply to initial diabetes patients. In addition, it is known from the literature that self-efficacy is an important predictor of health behavior (Stiecher, DeVellis, Becker, & Rosenstock, 1986); and many studies have aimed to explore

the correlation of social support and health behavior (Glasgow & Toobert, 1988; Hubbard, Muhlenkamp, & Brown, 1984). However, in the past, domestic studies on diabetes focused on disease cognition, attitude, compliance behavior, and the effectiveness of health education, but less on the impact of self-efficacy and social support on the self-care behavior of diabetic patients. In view of this, the author hopes that this study will explore the situation of self-care behavior of patients with non-insulin-dependent diabetes within two years, so as to provide an important reference for clinical caregivers in the early health education and intervention of early diabetes patients. Therefore, the purpose for (1) understand the early non insulin-dependent diabetes patients their self-care behavior, (2 I for the early non insulin-dependent diabetes patients their personal basic data, self efficacy, social support and self-care behavior correlation, (3), affect the early non-insulin-dependent diabetes patients with the important predictor of the self-care behavior.

Literature verification

Self-care is an important element in the successful management of a long-term chronic condition (Baker & Stem, 1993). For patients with chronic diseases, due to the characteristics and treatment of the disease, patients need special self-care, and effective self-care can not only successfully control the disease, but also avoid the progression and deterioration of the symptoms (Connelly, 1987).

In general, the main characteristic of chronic diseases is the long-term (long-term) of the disease, which is often complex and multifaceted, including taking medicine, drinking special, drunk wine, performing special health-related behaviors to monitor and evaluate the disease, improve happiness and tranquility, prevent complications, change habits and lifestyle to avoid risk factors (Connelly, 1987; Connelly, 1993). For patients with diabetes, outpatient treatment is a way to help patients control their disease, and patients have a daily tube of their health problems

The primary responsibility must be an active participant in the care process (Connelly, 1987; Connelly, 1993). Therefore, when caring for patients with chronic diseases, the role of caregivers is not only the provider of direct treatment, but also an educator, facilitator and supporter of effective self-care behavior (Connelly, 1987).

Studies have shown that women generally have better self-care behaviors than men (Mechanic & Cleary, 1980; Hubbard, Muhlenkamp, & Brown, 1984); When Lu Helin (Min 80) compared the effects of different health care methods on knowledge, attitude, behavior and blood sugar in diabetes patients, Found that the older the age, the better the self-care; Polly (1992) Study on health beliefs, self-care behaviors and glycemic control in 102 non-insulin-dependent diabetic elderly patients, There is a significant positive correlation between education level and self-care behavior; Hubbard, Muhlenkamp and Brown (1984) found that married elderly had better self-care than single ones. However, several studies still point to the self-care behavior and gender of diabetic patients (Hurley & Shea, 1992; Glasgow & Toobert, 1988; Wilson et aL, 1986; Bloom Cerkoney & Hart, 1980), age (Skelly, Marshall, Haughey, Davis, & Dunford, 1995; Hurley & Shea, 1992; Glasgow & Toobert, 1988; Wilson et al., 1986; Bloom Cerkoney & Hart, 1980), educational attainment (Wilson et al., 1986; Bloom Cerkoney & Hart, 1980), marital status (Bloom Cerkoney & Hart, 1980; Hurley & Shea, 1992), and sane status (Wilson et al., 1986) is not relevant. In terms of personal disease conditions, The domestic studies of Zhan (Min 72) and Lu Helin (Min 80) all found that The number of years of patients is positively correlated with their care behavior; However, some studies indicate the duration of illness (Hurley & Shea, 1992; Skelly, Marshall, Haughey, Davis & Dunford, 1995), the current treatment modalities (Glasgow & Toobert, 1988) and the presence of complications (Skelly, Marshall, Haughey, Davis, & Dunford, 1995) were not associated with self-care behaviors. Based izing the above literature, we can see the basic personal informationThere is no consistency in the correlation between the changes and self-care behaviors, so it is worth to discuss again.

Self-efficacy is derived from the social learning theory of Bandura, which includes two parts: efficacy expectation (efficacy expectations) and outcome expectation (outcome expectations). ° Efficacy expectation is the confidence that a person can successfully perform a behavior; and outcome expectation is a person to predict a result. Bandura Note that an individual's performance expectations are based on four different sources of information, namely behavioral performance outcomes (performanc accomplishm ents), alternative experience (vicarious experience), verbal persuasion (verbal persuasion), and emotional motivation (emotional arousal). Success in behavioral performance is the most reliable source of performance expectations, because it is based on personal experience, which can increase a person's expectation of their abilities. Alternative experience refers to the results obtained by observing the behavior of the demonstrator. When one evaluates his ability, one will use social

comparison and refer to the performance of others to improve the expectation of self-efficacy. Verbal persuasion is the easiest method, including the advice of others, health education or their own guidance, but the efficiency expectation caused by this method is often quite fragile and short, because it is based on no practical successful experience. As for emotional motivation, it uses attribution (i. e., direct cognitive change, the interpretation of emotional motivation reasons) and relaxation to correct escape behaviors. The emotions caused by the face of fear of things usually reduce the personal performance level of individuals and have a negative impact on their performance expectations (Bandura, 1977).

From the perspective of social learning theory, performance expectation is one of the determinants of prior behavior, which not only reduces anticipation, periodic fear and suppression, but also promotes the period of final success; it determines how much effort one will make and how long it can persist in the face of obstacles and aversion. In general, the higher the expectation of self-efficacy or success, the greater the effort, and the more persistent (Bandura, 1977).

Bandura Close efficiency expectations is self-efficacy, its prediction of behavior than expected, it to cause, individual engaged in a behavior starting decision, hard process, degree and in the persistence of the dilemma has a significant impact (Bandura, 1977) °, therefore, Bandura on the validation of self efficacy theory mostly focus on the efficiency of extensive expectations.

Strechcr et al. (1986) pointed out that self-efficacy is related to the change and maintenance of health behaviors, and can also be used as a predictor of health-related behaviors. At present, self-efficacy is widely explored in predicting health-related behaviors such as breast self-examination, smoking, drinking, exercise behavior, weight control, while the correlation between self-efficacy and self-care behavior in diabetes patients is still rare.

Many studies think that can be used help or emotional support, can affect a person's health and emotional, peace, and can prevent serious disease or stress consequences (Sherbourne, & Stewart, 1991) ° Orem (1991) has pointed out that social support is a kind of environmental resources, individuals can enhance its motivation in self-care behavior. Many studies have shown that social support is related to the degree of adherence to a health behavior (Glasgow, & Toobert, 1988; Haynes, Wang, & Gomes, 1987) ° Hubbard, Muhlenkamp and Brown (1984) also indicate a significant correlation between the degree of personal feelings of social support and the performance of special, positive health behaviors. Wilson et al. (1986) of 184 non-insulin dependent diabetes patients, with interpersonal support evaluation table (Interpersonal Support Evaluation List) and the social support for self-care for diabetes scale, as the two feeling society, support with often drunk, exercise, blood sugar test and drug drinking self-care

behavior. There are few studies on this area in China, and most of them only focus on discussing the impact of family support on behavior. For example, Zhang, Qiu, Xin and CAI (Min 80) pointed out that the better the family support of patients with diabetes, the better their compliance behavior is. However, some studies have shown opposite results. For example, Connelly (1993) started with 181 outpatient patients with chronic diseases, and the results showed that sensory social branches are negatively associated with generic, drunk self-care behaviors. As shown from the above literature, social support is generally considered to have a positive effect on self-care behavior; moreover, many social support scales mainly measure functionality, because many scholars believe that the applicable functional support is the most important level of social support (Sherbourne & Stewart, 1991). Therefore, this study will understand the relationship between it and self-care behavior from sensory functional social support.

noun definition

(i) Non-insulin-dependent diabetes patients at the initial stage: Because diabetic patients are in terms of blood glucose control and learning, it takes a period of time to become stable and familiar. Therefore, the author is based on clinical practice and discuss with senior clinical physician, the so-called early non-insulin dependent diabetes patients as patients by specialist diagnosed as non-islets, element-dependent diabetes, until the day, the disease for more than six months and not more than two years.

(ii) Self-care behavior: refers to the actual behaviors of monitoring, planning, and performing daily life care of diabetes, including: drunk food, exercise, drug and blood glucose monitoring, foot care, and prevention and treatment of high and low blood glucose. This study was measured by the Diabetes Self-care scale developed by the revised Hurley and Shea (1992).

(iii) Self-efficacy: refers to the degree to which the subjects monitored, planned and performed daily care of diabetes. This study was measured on the insulin-treated diabetes self-efficacy scale developed by the revised Hurley and Shea (1992).

(iv) Social support: refers to the potential functional support that can be used by individuals, including: relatives, friends, neighbors, residents, medical staff and other important others in terms of emotional, information and substantive support. This study was conducted by revised Sherbrourne and Stewart (1991).

method

one, Study design and object

[9]The diabetes patients in metabolism and endocrinology in the Kaohsiung area should meet the following criteria: (1) diagnosed as non-insulin-dependent diabetes (& ype II); (2) had disease for more than six months and no more than two years; (3) was over 18 years old; (4) aware, using words, and communicating with medical staff; (5) After the investigator explained the study purpose, the case is willing to participate in the study. The study was collected from December 10,2085 to February 26,2086. Excluding questionnaires with incomplete data, a total of 130 valid samples were obtained.

two, research tool

This study collects data with a structured questionnaire, which includes:

(-Basic information of the patient: This part mainly collects the demographic and disease information of the cases, including: sex, sex, age, marital status, religious belief, education, occupation, social status, period of illness, current treatment, presence of other diseases and hospitalization experience, etc.

(ii) Diabetes Self-care Scale: Diabetes Self-care Scale (Diabetes Self-Care Scale developed by Hurley and Shea (1992), DSC). The original scale, which includes 28 questions, including general, drunk food, exercise, foot care, blood glucose and urine sugar monitoring, and insulin injection, is scored with six points. With the consent of the original author, the authors referred to other domestic and foreign materials (Crabtree, 1986, Xiao et al., Min 83; Zhang, Lin, Min 86) and revised them according to the characteristics of the subjects, and finally became the structural self-prepared scale of 27, using the Likert five-point scoring method for the subjects, from 1 to 5 points to represent completely failed, rarely done, sometimes done, frequently done, and completely done, the higher the score indicates the better the self-care behavior of diabetes.

(iii) Social support: compiled from the translation of social support (Medical Outcomes Study (MOS) Social Support Survey) developed by Sherbrourne and Stewart (1991). This questionnaire is applicable to patients with chronic diseases, and mainly measures the potential functional support felt and received by individuals. The so-called work and performance support refers to the degree of special functions existing between people. Original questionnaire a total of 20 questions, respectively contains emotional / information support, substantial support, positive club, interaction, emotional support and a question about social support, structural indicators, take five points, subjects were asked about false, if they have need, its available support, score from 1 to 5 points, respectively represent never, rarely, sometimes, often, always, the higher the score said the higher the social support. After obtaining the consent of the original author, the researcher deleted the first question and became the Likert fifth grade scale of the 19 questions.

(iv) Diabetes Control Self-efficacy Scale: The study tool was translated and modified as the insulin-treated Diabetes Self-efficacy Scale (Insulin Management Diabetes Self-Efficacy Scale, IMDSES) developed by Hurley and Shea (1992). The original scale consists of 28 questions, including general, drunk food, exercise, foot, mental care, blood glucose and urine sugar monitoring, and insulin injection, scoring in the same way as the self-care scale. This study revised it to 27 questions, and changed it to Likert five-point scoring method, the subjects were selected according to whether they could do it, from 1 to 5 points, respectively representing the completely uncertain, 20 or 30% sure, 50% sure, 70% sure, very sure. In order to make it easier for the subjects to answer, the negative questions were changed to positive questions, and the total score is the cumulative score, the higher the score, the higher the self-efficacy.

Third, reliability and validity

The two-week retest reliability analysis of 30 cases, and the retest reliability of diabetes self-care scale, social support scale and diabetes control self-efficacy scale was 0.96. In the analysis of intrinsic consistency, at the time of the formal test, the SA Cronbach, the sa coefficients were 0.82,0.95, and 0.87, respectively.

This institute used ″diabetes self-care scale″, ″social support questionnaire″ and ″diabetes control, system self efficacy scale″ are compiled by English original scale translation, because the three scales for local samples, based on the research object, national conditions and culture, is 130 extensive sample data factor analysis, to verify ″diabetes self care scale″, ″social support survey, table″ and ″self efficacy of diabetes control scale″ construct validity. The KMO value (Kaiser-MeyerOlkin Value) of the three component scales was 0.68,0.92 and 0.75, respectively, All are close to 0.70, The selection is appropriate and suitable for factor analysis ° before main component analysis (principal components method), The number of factors is selected by the characteristic value (eigenvalue) greater than 1 and the reference factor steep order diagram (factor scree plot); And the factor load (factor loading) is greater than 0.30 as the topic condition..00

A total of 26 questions were retained in the Diabetes self-care scale, and the question ″I regularly go to the outpatient department for medical treatment and take medicine″ was deleted. Perhaps, because the study was collected in the outpatient department, the vast majority of cases fully achieved this behavior. In factor analysis, extract five factors, named as movement, I drink, medicine, self care, medicine, foot self care and high and low blood glucose prevention and self care, each factor contains the problem, items, factor load, features and interpretation are detailed in the table
—0

Three factors were selected in the social support questionnaire, and all the 19

questions of the original scale were retained. The three factors were named emotional support (11 questions), information support (4 questions) and substantive support (4 questions), and the variation explained was 59.0%, 9.4% and 6.3%, and the total explained variation was 74.7%.

A total of 26 questions were retained on the Diabetes Control Self-efficacy Scale, and five factors were extracted. Drunk drunk, self-efficacy (question 6), exercise self-efficacy (question 4), foot self-efficacy (question 5), drug and glucose monitoring self-efficacy (question 7), and hypoglycemic prevention and treatment self-efficacy (questions 4), explain 26.0%, 13.4%, 10.5%, 8.8% and 5.6%, explaining 64.4% of the total variation.

Iv. Analysis of the data

All data shall be processed by computer and analyzed with SPSS / Windows and set software. The statistical methods used are: (1) Personal basic data is described by the distribution of times and the percentage.(2) Self-care behavior, social support, and self-efficacy scores are described by frequency distribution, percentage, average value, standard deviation, etc.(3) The relationship and relative importance of one-way ANOVA), Pearson product difference correlation analysis (Pearson's product moment correlation) and stepwise regression analysis (stepwise multiple linear regression).

bear fruit

1. Basic data of the studied subjects

A total of 130 individual cases were collected in this study, As shown in Table 2, 82 (63.1%), 48 males (36.9%); Age ranged between 26 and 80 years, At 41 to 60 (54.7%), The mean age was 55.3 years, Standard deviation is 11.8; The marital status was married in the majority (83.8%); In terms of religious belief, Most of the cases (86.2%) were religious; The education level of the cases was mostly below the primary school (67.7%); In terms of the employment situation, 68 (52.3%) cases were unemployed, 62 bits (47.7%) are currently employed; Social and economic status is based on education level and occupation, Divided into grades I to V, The more the series is, the lower the social status (Lin, Min 67), Most cases were of grade V, For 70.8%.

Table 1 Factor analysis of the Diabetes Self-care Scale

Factor category and the topic	Factor load	Feature	Interp
Factor I: The lotus movement takes good care of the		5.68	21.
No matter how busy I am, I will take the time out	.94		
I usually keep regular exercise (more than 3 times	.93		
When I don't want to exercise, I still exercise for	.91		
Even on the weekend holidays, I still do sports.	.89		
Factor II: Diet rhymes with itself		3.61	13.9
When I go out to a familiar place to dinner (such Dietary principles of urinary disease.	.90		
When I dine in the unfamiliar place, I still follow	.89		
I drink diabetes, even at meals.	.79		
Even on holidays, birthdays or outings, I drink	.79		
I do drink drunk in the same kind of food.	.68		
I eat on time every day.	.32		
Factor] II: Drug and blood glucose monitoring for		2.33	9.0
I usually take diabetes medication at the time	.72		
When I was out, I still took my diabetes medication	.70		
I took diabetes drugs at the dose given by the	.61		
When I was out, I still tested my blood sugar or urine	.60		
I usually measure blood sugar or urine sugar at	.55		
I recorded the results of each blood sugar or urine	.36		
When my body is not well, I take blood sugar or urine	.34		
Factor IV: a strict foot level of self-discipline		2.10	8.1
I usually wear appropriate shoes and socks,	.84		
I still wear proper shoes and socks when going	.76		
I checked my feet or applied ointment or lotion on	.50		
I trim my toenails by diabetes care.	.36		
When there are problems with the foot (for example, Find a doctor to deal with.	.31		
Factor V: Prevention and treatment of self-care of high		1.58	6.1
When blood sugar control is poor (such as staying handle.	.72		
When my blood sugar is too low (such as: cold sweat, Take the correct self-treatment measures (such as:	.56		
When my essence becomes higher than normal (like; I immediately took the right self-handling	.55		
When I exercise, I follow medical advice to prevent	.43		
amount to			58.

The time of the study was calculated based on the patient Other diseases; and none of the subjects were most recent

Specialist diagnosed non-insulin dependent diabetes, Experience o

By the end of the interview, the study results showed Self-care behavior of ,

All were 15.9 months with standard deviation of 6.80, more

One 24 months; treatment options for current glycemic control,

To take oral hypoglycemic drugs and diet, exercise to control Divide the average

(93.8%); in the presence of other diseases and nearly three The high possible score,

In terms of hospitalization in November, 42 people (32.3%) were killed in Table 3, and

The mean score was 84.09 (64.69%), which was similar to the study of Polley (1992), indicating that the score index of non-insulin-dependent diabetes mellitus was 70%. Therefore, it shows that diabetic patients need to strengthen the implementation of self-care behavior.

In terms of sub-items, the score of "drug and blood glucose monitoring self-care" was significantly higher than other sub-items, 81.32%; while the score of "prevention of high and hypoglycemia and handling self-care" was the lowest, only 49.88%;

Table 2. Basic information of (N=130)

Item S	nu	percentag
sex		
man	48	36.9
Female age	82	63.1
26—4. year	15	11.5
41 to age 60	71	54.7
Marital status of those over 61 years old	44	33.8
Unmarried, divorced, or	21	16.2
Married religious beliefs	10 9	83.8
not have	18	13.8
Yu education level	11 2	86.2
Uneducated	47	36.2
primary school	41	31.5
National (early)	9	6.9
High school (job)	18	13.8
	8	6.2
university	5	3.8
Institute or above	2	1.5
occupation		
No, family management, has	68	52.3
Have social status	62	47.7
I	2	1.5
II	6	4.6
III	12	9.2
IV	18	13.8
	92	70.8
The death period		
Half year 1 year (7-12	53	40.8
1 year or 2 years (13-24 months)	77	59.2
Oral hypoglycemic drugs +	12	93.8
Injection trypsin + diet and exercise insulin + oral	2	1.5
Diet and exercise, without other diseases	6	4.6
in compliance with E	88	67.7
Hospital admission in the last three months	42	32.3
not have	13	100.0
have	0	0.0

The lower scores were "exercise self-care" and "foot self-care", with scores of 57.19% and 57.05%, respectively (see Table 3).

(ii) Social support and self-efficacy

In terms of social support, the subjects scored 69.70%; the highest score was "substantive support", followed by "informative support" and "emotional support", indicating that the case felt the least emotional support from important others (see Table 3).

In Table 3, the score was 77.45%, the highest score was 90.26% for the self-efficacy, and the patient had less confidence in performing exercise self-care.

3. The relationship between basic data, self-efficacy, social support and self-care behavior in diabetic patients

(-) Analysis of basic personal data and self-care behavior When exploring the differences in self-care behavior, For the analysis required, Dividing educational attainment into three groups, That is, below primary school, national (primary) and high (vocational), college or above into a group; While social status grades I, II and III are classified as middle social status above, Class IV is social status, Level V is low social status, To make this classification for analysis; And because the number of cases treated with oral hypoglycemic drugs and insulin is too different, Case current treatment modalities will not be listed as data for the following statistical analysis. The results showed that age, period of illness and self-care behaviors; gender, marital status, religious belief, education level, occupation, social status, and self-care behaviors (see Table 4).

(ii) Relationship between self-efficacy and self-care behavior

Table V shows a significant positive correlation between the total self-efficacy performance and the total self-care behavior; since the corresponding self-care performance, the better the self-efficacy, the better the self-care behavior.

Table 3 Scores of self-care behavior, social support and self-efficacy (N=130)

« §	Average value, standard deviation	Score
Total amount of self-care table	84	
Diet self-care	19	
Exercise self-care	11.44	
Drug and blood glucose monitoring for	28.46	
Foot self-care	14.26	
High and low prevention of hypoglycemia and	9.98	
Total table of social support	66.22	
Emotional support	36.27	
Information support	13.84	
Substantial support	16.11	
Total volume table of self-efficacy scale	100.69	
Diet self-efficacy	22.35	
Exercise self-efficacy	12.89	
Drug and blood glucose monitoring for	31.59	
Foot self-efficacy	19.49	
The prevention and treatment of high and	14.36	

45.5.3.3.3. 7.:6 00.07,10.40 ▪.24.99.05.89.27

'1 4551422269781) 70941954 3.0.8 9 19 2 5 8

Note: Score index = (average score / highest possible score) X100%

Table Table Single-factor variation number analysis of basic data and self-care behavior (N = 130)

The r coup le

cen tre

Below> Degree 'f alling-rising tone

In

Ground and lower ••

No medium low,no illness

The vocational

82 48 2109 2715 88 62 68 92 18 20 42 88

3 3 07 :09 3 • 9 785 3 & 15 4 03 92 28 70 81 70

413.05 .81 60 11 .95 514.73 29 83 88 .02 9 3

2 8 .00 .01 ,00 .91

(It will support the relationship with self-care behavior

According to Table 6, social support scores showed a significant positive correlation with self-care behavior scores, that is, increasing social support Good, the better their self-care behavior is. In addition, emotional support, informative support, and substantial support are significantly positively associated with drunk self-care, emotional support, information, and sex

-#-70

Support was positively associated with self-care behaviors such as prevention and management of high and hypoglycemia.

4. Important predictive variation of self-care behavior in diabetes

In order to explore the important variables in predicting self-care behavior, the variables in the study were transformed into parts of category variables and transformed into virtual variables (dummy variable) ° . Before stepwise regression analysis, all the variables were analyzed by the correlation matrix and collinearity and independent judgment. Self-care behavior was taken as the change term, and self-efficacy and social support were included respectively. After the change, it was found that the important factor affecting self-care behavior was ″self-efficacy″, which can explain 74.0% of the total variation in self-care behavior (see Table 7 for details).

1. Self-care behavior of diabetic patients

In this study, the score of self-care behavior was only 64.69%, indicating that the patient did not perform well. In the

Table V, correlation analysis of self-efficacy and self-care behaviors (N=130)

Self-care, and self-efficacy	Total amount of table	food and drink	movement	Drugs and blood	foot	Prevention and treatment of
Total amount of table	.865***	.649***	626***	393***	556***	.219*
food and drink	.699***	.907***	238**	.343***	.213*	.304***
movement	.593***	.240**	930***	.102	.272**	-.100
Drugs and blood glucose	.552***	342***	181*	.731***	.384**	.028
foot	.528***	,244**	350***	.205*	.750**	one.004
Prevention and	.439***	.296**	122	.003	.403**	.623**

*p<0.05 **p<0.01 ***p<0.001

Table VI Analysis of the correlation between social support and self-care behaviors

Self-care, and	Total amount of table	food and drink	movement	Drugs and	foot	Prevention and treatment of
The metric	.320***	.275	.118	.007	.273**	.326**
emotional	.318***	.244	.138	-.004	.289**	.325**
Informati	.257**	.281	-.008	-.071	.247**	.413**
materiali	.235**	.213	.132	.101	.132	.101

*pV0.05 **p<0.01

***p<0.0

Table 7 Stemental regression analysis of self-care behaviors (N=130)

variable	Raw regression coefficient (B)	Standardized regression	Cumulative coefficient of	F
self	0.807	0.862	0.740	368.491***
constant	2.876			

***p<0.001

self-care behavior, self-care best with drugs and blood glucose, which is similar to the results of zhang, qiu, xin, CAI (min 80)> Polley (1992), Anderson, Fitzgerald and Oh (1993), this may be due to taking diabetes drugs, and blood glucose monitoring is diabetes must perform routine activities. In addition, this study case in the high and hypoglycemia prevention and treatment of self-care do the worst, may be therefore since, I care behavior and the case of knowledge, experience, conscious seriousness, so it is easier to be neglected, because, the future diabetes education should emphasize the aspect of knowledge, judgment and importance.

Ii. Social support and self-efficacy of patients with diabetes mellitus

Individuals with long-term health problems are prone to high risk of social isolation and social interaction disorders, and social relationships are often prone to confusion and isolation under the pressure of their chronic diseases and related treatments (Tilden, & Weinert, 1987). From the social support of the subjects in this study, substantive support is the best; followed by information support and the worst by emotional support. The main reason may be that substantive support is easy to feel and based to the national conditions, Chinese people express their emotions conservatively, so that the case feels less emotional support. Therefore, in the process of diabetes care in the future, medical staff and important patients, in addition to providing substantial diabetes disease, information and information support, should give patients more care, trust and timely express their love or love to them.

In the case of self-efficacy in diabetes control, the study sample was the most confident and confident of the study sample, with 118 African American women with non-insulin-dependent diabetes, and found the best self-efficacy in medication and blood glucose monitoring, and the worst drunk food and exercise self-efficacy. This may be due to the clear goal of drug and blood glucose monitoring, and the arrangement and continuity of exercise time may need to overcome more obstacles, so it is less confident.therefore, Clinical caregivers can design and develop the four sources of self-efficacy proposed by Bandura (1977): oral persuasion, emotional motivation, alternative experience and behavioral performance results, For example, Lian used oral persuasion to teach individual cases about the importance of exercise therapy to diabetic patients and the matters needing attention; Based on the different background information of the patient, Jointly formulate a gradual exercise and self-care plan, Change the wrong cognition and escape behavior of the execution of motor planning by emotional motivation; Share the experience with them; and then encourage the case to experience the benefits of exercise to enhance the results of behavioral performance, To increase the confidence of individual cases in performing exercise self-care behaviors, Make it think that as long as they redouble their efforts to hold the ®, It will certainly overcome the

-# -73

obstacles.

3. Factors affecting the self-care behavior of diabetic patients

The self-care behavior of this study did not differ significantly from the basic data explored, which shares the same findings as many studies (Hurley & Shea, 1992; Wilson et al., 1986; Glasgow & Toobert, 1988; Skelly, Marshall, Haughey, Davis, & Dunford, 1995)。Social support table only with drunk food, foot, and high and low blood sugar prevention and processing of self care behavior significant positive correlation, and Wilson et al. (1986) for 184 non-insulin dependent diabetes patients extensive found, the social support and drunk food, exercise, blood sugar monitoring, drugs and other self-care results are not the same. As for the subscale, the better the emotional, information, substantive and other social support, the better the self-care behavior, the better, it is difficult to compare with other studies. It can be seen from the study results that although the case scored the highest support in the substantive society, the correlation between social support and self-care behavior is high. In addition, regardless of the social support scale or the subscales, and movement, drug and blood glucose monitoring, it may be due to the self-care behavior in these two aspects, the implementation goals are clear, so the case requires less need for emotional, informative and substantive support from others. Thus, in the future health education process of diabetes, except

In addition to providing appropriate social support for patients, it can also be used to improve the implementation of self-care activities for different functions, especially the social support in both emotional and informative aspects.

Self-efficacy was significantly positively associated with self-care behavior, and two other scales. Moreover, it can be seen from the study results that self-efficacy is an important factor in predicting self-care behavior, while social support has a significant correlation with self-care behavior, but it is not an important predictor of self-care behavior. Therefore, although the influence on self-care behavior is multicausal, it is very important for early non-insulin-dependent diabetes patients in the face of complex treatment planning and self-care planning.

propose

(~ Into the nursing practice aspect

1. Understanding the self-care behavior of early non-insulin-dependent diabetes patients can be a reference for future diabetes health education:

(1 In diabetes self-care behavior, the awareness of hyperglycemia and hypoglycemia and the importance of this behavior.

(2) To confirm and provide appropriate social support, especially to give more emotional support such as patient need, caring, and listening, to enhance the self-care behavior of diabetic patients.

(3) Assess the degree of patient self-efficacy for diabetes control, explore the reasons for low self-efficacy, and increase the confidence in performing diabetes self-care activities from the four sources of Bandura (1977).

2. The "Diabetes Self-care Scale", "Social Support Adjustment Scale" and "Diabetes Control, Self-efficacy Scale" are used as tools for clinical caregivers to assess the home self-care situation, social support and self-efficacy for outpatient non-insulin-dependent diabetes patients.

(ii) In terms of nursing research

1. The research tools and results of this study can be used by relevant domestic researchers in the future.

2. The factors affecting health behavior are multifactors, and in future studies, other prediction variables such as self-description, reading, health belief and health motivation can be included in the studies to explore the importance of these variables to self-care behavior.

Research restrictions

(i) This study is not a random sampling method, and is limited to non-insulin-dependent diabetes patients in a medical center in Kaohsiung, which makes the study restricted from inference to all initial non-insulin-dependent diabetes patients.

(ii) The cross-sectional study in this study fails to better understand and observe the changes of patients' self-care behavior. If it can track them for a long time, we can better understand the different effects of various factors on self-care behavior of

diabetes.

Thank you

Thank kaohsiung medical hospital attached and memorial hospital metabolism, endocrine internal medicine XinXiZhang attending physicians and sugar urine, disease WeiJiao nursing teacher assist in the case, Yang ming university Lin, aunts, associate professor, kaohsiung medical institute yong-yuan zhang correction on the content, and 130 diabetes, make this research successfully completed, sincerely to thank.

reference documentation

Lin Xian (Min 67) Community Mental health Taipei: Buffalo.

Zhang Fenghui, Qiu Qirun, Xin Xizhang, CAI Ruixiong (Min 80) Research Manager of cognition, Attitudes, Compliance Behavior, and Control of haemochrome, 38 (3) 59-69.

Zhang Fenghui, Lin Qiuju (Min 86) -Self-care of Insulin-dependent Diabetes Patients by self-efficacy Journal Medical Science, 13 (6), 351-359.

Zhan Hui-min (Min 72) -Discussion of the related treatment of diabetes patients in Taipei, 10 (3), 256-272.

1 ˆ Mei xiu and Lin Qiuxiang (Min 80) compare the effects of group health education and na other health education on knowledge, attitude, behavior and changes in blood glucose care in diabetes patients, 38 (4), 101- -112.

Dai Shuyuan (Min 82) Treatment of diabetes in the elderly negative wan Medical Community, 36 (6), 25-27.

- -Lizhen, Chen Yuezhi, Cai Shize, Lin Ruixiang, Li Yanjin, Xuan Liren, Dai Dongyuan (Min 83) -Self-care of insulin-dependent diabetes patients and the impact on diabetes control ■ Zhonghua Wei Zhi, 13 (3), 276-284.

Anderson, R.M., Fitzgerald, J.T., & Oh, M.S.(1993).The relationship between diabetes-related attitudes and patients' self-reported adherence.The Diabetes Educator, 19(4), 287-292.

Baker,C., & Stern, P.N.(1993).Finding meaning in chronic illness as the key to self-care.Canadian Journal of Nursing Research, 25(2), 23 — 36.

Bandura, A.(1977).Self-efficacy: Toward a unifying theory of behavioral change.Psychological Review, 84(2).191-215.

Bloom Cerkoney, K.A., & Hart, L.K.(1980).The relationship between the health belief model and compliance of persons with diabetes mellitus.Diabetes Care, 3(5), 594-598.

Connelly, C.E.(1987).Self-care and the chronically ill patient.Nursing Clinics of North America, 22(3).621-629.

Connelly, C.E.(1993).An empirical study of a mode) of self-care in chronic illness.Clinical Nurse Specialist, 7(5), 247-253.

Crabtree, M.K.(1986).Self-efficacy and social support as predictors of diabetic self-care.Unpublished doctoral dissertation, Unversity of California, San Francisco.

Glasgow, R.E., & Toobert, D.J.(1988).Social environment and regimen adherence among type II diabetic patients.Diabetes Care, 11(5), 377 — 386.

Haynes, R.B., Wang, E., & Gomes,

M,D.(1987).A critical review of interventions to improve compliance with prescribed regimens.Palienl Education and Counseling, 10, 155 — 166.

Hubbard, P., Muhlenkamp, A.F., & Brown, N.(1984).The relationship between social support and self-care practices.Nursing Research, 33 (5), 266 — 270.

Hurley, C.C., & Shea, C.A.(1992).Selfefficacy: Strategy for enhancing diabetes self-care.The Diabeles Educator, 18(2), 146 -150.

Peveler, R.C., Davies, B.A., Mayou, R.A., Fairburn, C.G., & Mann, J.I.(1993).Self-care behaviour and blood glucose control in young adults with type I diabetes mellitus.Diabetes Medicine, 10, 74-80.

Polly, R.K.(1992).Diabetes health beliefs, selfcare behaviors, and glycemic control among older adults with non-insulin-dependent diabetes mellitus.The Diabetes Educator, 18(4), 321 _ 327.

Sherbourne, C.D., & Stewart, A.(1991).The MOS social support survey.Social Science and Medicine, 32(6).705-714.

Skelly, A.H., Marshall, J.R., Haughey, B.P., Davis, PJ„ & Dunford R.G.(1995).Self-efficacy and confidence in outcomes as determinants of selfcare practices in inner-city, African-American women with non-insulin-dependent diabetes.The Diabetes Educator, 21(1), 38 _46.

Irvine, A.A., Saunders, J.T., Blank, M.B., & Carter, W.R.(1990).Validation of scale measuring environmental barriers to diabetes-regimen adherence.Diabetes Care, 13(7), 705—711.

Mechanic, D., & Cleary, P.D.(1980).Factors associated with the maintenance of positive health behavior.Preventive Medicine, 9, 805 — 814.

Orem, D.E.(1991).Nursing: Concepts of practice (4th cd.).St.Louis:Mosby-Ycar Book, Inc.

Strecher, V.J., DeVellis, B.M., Becker, M.H., & Rosenstock, I.M.(1986).The role of self-efficacy in achieving health behavior change.Health Education Quarterly, 13(1), 73_91.

Tilden, V.P., & Weinert, C.(1987).Social support and the chronically ill individual.Nursing Clinics of North America, 22(3), 613 _ 620.

Wilson, W., Ary, D.V., Biglan, A., Glasgow, R.E., Toobert, D.J., & Campbell, D.R.(1986).Psychosocial predictors of self-care behaviors (compliance) and glycemic control in non-insulindependent diabetes mellitus.Diabetes Care, 9(6), 614 -622.

Wooldridge, K.L., Wallston, K.A., Graber, A.L., Brown, A.W., & Davidson, P.(1992).The relationship between health beliefs, adherence, and metabolic control of diabetes.The Diabetes Educator, 18(6).495 - 500.

Self-Care Behaviors and Related Factors in Outpatients Newly Diagnosed with Non-Insulin-Dependent Diabetes Mellitus

J.S.Wang • R.H.Wang • C.C.Lin

ABSTRACT

The purpose of this correlational study was to understand self-care behaviors and related factors in outpatients newly diagnosed with non-insulin-dependent diabetes mellitus (NIDDM).Data were collected by purposeful sampling from 130 outpatients with NIDDM in a Kaohsiung medical center from December 10 1996 to February 26 1997. The results indicated: (1) the preventing, treating high and low blood sugar reactions subscale of self-care behaviors, which had the lowest proportional scores, was only 49.88%; (2) social support and self-efficacy scores correlated significantly with self-care behaviors; (3) according to a multiple stepwise regression analysis, self-efficacy was found to explain 74.0% variance of self-care behaviors.The results of this study could be used as a reference for further diabetes health education and as a suggestion for future research.

Key words: self-care behaviors, self-efficacy, social support, non-insulin-dependent diabetes mellitus.
