# Supplementary material for: Efficacy of a smartphone application for helping individuals with type 2 diabetes mellitus manage their blood glucose: a protocol for factorial design trial
Source: Trials. 2023 Jul 22;24:468. doi: 10.1186/s13063-023-07489-5 (PMC10362696; doi:10.1186/s13063-023-07489-5)
Supplement: Supplementary file 6 — Additional file 6. [file 13063_2023_7489_MOESM6_ESM.pdf]

·specialist consensus·

Statistical considerations for sample size determination in a clinical trial

CCTS Working Group, Chen Bingyan (written)

Sample size determination (sample size determination), also known as sample size estimatio n (sample size estimatio n), refers to the required sample size calculated to meet the statistical accuracy and reliability (guarantee of control and inspection efficiency of class I errors)，It is a very important link in the clinical trial design，Direct related to the reliability of the study conclusions、repeatability，And the efficiency of the research。The sample size estimate is also a cost-The trade-off process between effect and test efficacy. ICH E 9 (1998) stated that the imminent

The sample size for the bed test must be large enough，To reliably answer the relevant questions raised by the research hypothesis；At the same time, it is not too big and cause waste。The method for estimating the sample size should be elaborated in the study protocol，Including the parameters on which the sample size was calculated，Such as variance、mean、reaction rate、Prevalence of positive events、Differences, etc。This paper is applicable to the confirmatory testing。

The main factor to be considered in the sample size estimation

After determining the purpose of the clinical study，First, consider the trial design，Including the selection of control (e. g., standard control, positive control, placebo control, dose control, etc.), comparison type (e. g., superiority test, non-inferiority test, equivalence test), design type (such as parallel design, cross design, design, factorial design, group sequential design, etc.), main indicators (quantitative, qualitative, survival time), etc.; secondly, considering the statistical analysis method, And propose the assumption of effect size (effect size); then define the statistical characteristics according to the test characteristics, Such as statistical distribution, test level (significant level), test efficacy (power), single two-sided and allocation proportion, etc.; then apply the correct sample

The quantity estimation method calculates the sample size；Finally, according to the covariates、Aof rate in the test、The elimination rate and compliance should be adjusted appropriately。

1. Study objective and trial design

(1) Study purpose

In terms of clinical trials，In the confirmatory study, the study purpose is mainly reflected in the effectiveness evaluation and safety evaluation。Sample size estimation is often used for validity evaluation。

(2) Comparison types and their test hypotheses

The common comparison types of clinical trials include superiority test (superi-ority trial), equivalence test (including bioequivalence test) (equi v-alence trial), and non-inferiority test (non-inferiority trial), etc. The comparison of the two groups of effect sizes is taken as an example，setting

Chinese clinical trial biostatistics group members (sorted by name pinyin): Chen, yan, Chen Qiguang, Chen qiguang, he jia, Huang Qin, Jin Pihuan, li kang, li ning, li wei, li, LingLi, Liu Yuxiu, Su Binghua, sun gao, Wang Wubao, wang tong, wei zhaohui, summer knot, yao, yao, morning, yi, east, Yin, flat, in, hao, Zhang Luo diffuse, zhao qing.

(C)1994-2022 China Acad■mic Journal Electronic Publishing House. All rights reserved. http://www.cnki.net

Excellent effect、The bounds for equivalence and non-inferiority are Δ，Explain their test hypothesis and inference conclusions。

Optimality test：The purpose of the trial is to verify whether the test group effect is better than that of the control group，If the study does not set a superiority threshold，The test hypothesis is that:

$H_0: \mu_T = \mu_C$；

$H_1: \mu_T \neq \mu_C$。

If P α, and $\bar{X}_T > \bar{X}_C$, It can be inferred that the test group is better than the control group。here，μ_TAnd μ_CRepresents the overall mean of the test and control groups, respectively,$\bar{X}_T$And$\bar{X}_C$Sample means representing the test and control groups, respectively.

If the study superiority threshold is Δ (Δ> 0, the same below), namely strong superiority, the test hypothesis is:

$H_0: \mu_T - \mu_C \leq \Delta$;

$H_1: \mu_T - \mu_C > \Delta$ 。

At this point, if P α, it can be inferred that the test group is better than the control group.

Equivalence test：The aim of the trial was to verify that the test group effect was comparable to the control group。If the study set the equivalent threshold value Δ，The test hypothesis is that:

$H_0: \mu_T - \mu_C \leq -\Delta$ perhaps $\mu_T - \mu_C \geq \Delta$;

$H_1: -\Delta < \mu_T - \mu_C < \Delta$ 。

This is the same case for the upper and lower limits。If the lower limit is for Δ_1With upper limit Δ_2Different, then the test hypothesis is:

$H_0: \mu_T - \mu_C \leq -\Delta_1$perhaps $\mu_T - \mu_C \geq \Delta_2$;

$H_1: -\Delta_1 < \mu_T - \mu_C < \Delta_2$。

At this point, if P α, the efficacy of the test group can be inferred as equivalent to that of the control group.

Non-inferiority test：The purpose of the trial was to verify whether the test group effect was not inferior to the control group。If the study set a non-inferiority threshold of Δ，The test, assuming that:

$H_0: \mu_T - \mu_C \leq -\Delta$;

$H_1: \mu_T - \mu_C > -\Delta$ 。

At this point, if P α, it can be inferred that the test group is not inferior to the control group.

(3) Design type

The design model of clinical trials is relatively simple，The common one is a parallel design、cross-over design、factorial design、sequential design、Group random design and adaptive design, etc。Detailed details of these designs is refer to relevant literature.

2. Main indicators

(C)1994-2022 China Acad■mic Journal Electronic Publishing House. All rights reserved.   http://www.cnki.net

The sample size of clinical trials is usually estimated based on the corresponding assumptions of the main indicators. In phase II and III clinical trials, the main index is generally the effectiveness evaluation index, while the main index of post-marketing phase IV clinical trials can be the effectiveness evaluation index，It can also be a safety evaluation index，Or both。If the sample size estimation should be based on both the primary efficacy indicators and the primary safety indicators，Statistical assumptions should be designed for effectiveness, sex and safety respectively，Sample size was calculated one by one，The final sample size was taken as the largest sample size。The main indicators should be clearly defined in the study protocol，Usually determined based on expertise，It should be a indicator of consensus or recognition in the professional field，Generally derived from a certain standard or guide，Or from the authoritative published works or expert consensus in the professional field。Main indicators should not be too much，There is generally only one。When there are more than one primary indicator，Sample size estimates consider the multiplicity problem of hypothesis testing。

In the process of defining the main indicators，It is not just the meaning of the indicator，Its measurement time point、The measurement means and calculation methods shall be indicated。The type of indicators should be clear，This point is very important，Because of the sample size estimate and the number According to the analysis needs to follow this。for instance，Certain indicators can have a quantitative value、Qualitative (such as effective and invalid), grade (such as recovery, obvious, effective, effective, effective, invalid), survival time and other different types. Corresponding to the different types of indicators, the sample size estimation method is also different。so，The definition of the main indicators in the scheme should be specific to the indicator type。

3. Effect size

The effect size is one of the most important parameters required for the sample size estimation，According to the different indicator types，The common effect sizes are：Group difference or standardized difference of mean, group difference or ratio (RR, HR), OR, or correlation coefficient of rate、Regression coefficients et al。

The effect size parameters were determined mainly based on the following three pathways：

(1) Results of any previous studies of this project. That is, the results of pretrials, exploratory trials (phase I or phase II clinical trials), and single-center trials derived from the same project are used as the basis for determining the parameters。As such research findings are internal evidence，And therefore is the preferred pathway。

(2) Based on the research results of others. When previous studies in this project could not provide exact parameter data，Or when the study has not yet been conducted，Parameters can be determined based on publicly published findings。Because such research findings are external evidence，So it is a secondary route。If more than one similar study is published publicly, it is best to take the combined effect size obtained by meta-analysis as the parameter of the sample size estimation。

(3) Based on the expected results of this trial. If no results of previous studies can be used (on their own or others), or the data of previous studies cannot obtain the parameters required for this trial design (such as the data of previous studies are from two parallel group designs), it can be preset in the expected form，Usually expressed as a generalized effect size。With sufficient confidence in the test drug or device, the expected effect size is large (if set to 0. 8) and the required sample size is small。Insufficient confidence in the test drug or device，Then the expected effect size is small

(C)1994-2022 China Acad■mic Journal Electronic Publishing House. All rights reserved. http://www.cnki.net

(if set to 0. 2), and the sample size required is large. Confidence in the test drug or device is acceptable，The expected effect size is moderate

Level (if set to 0. 5), the required sample size is also medium size at this time.

besides，For single-arm designs or paired-pair two-arm designs，If the determination of standard control parameters (or target values) is involved, the priority order of the approach is roughly international standards, national standards, industry standards (including guidelines, etc.), enterprise standards recognized by the authorities, and external evidence (comprehensive results of similar studies, such as meta, analysis results).

4. Statistical characteristics

The statistical characteristics of sample size estimation are mainly statistical distribution、inspection level、power of test、Single-bilateral and balanced or not, etc。

statistical distribution：The choice of the sample size estimation method is closely related to the assumed statistical distribution of the main indicators，A parameter method is chosen based on a normal distribution，A non-parametric approach is chosen based on a non-normal distribution。Similarly, the sample size estimation method for survival analysis varies by the assumption of the Weibull distribution family。

Inspection level: the inspection level is also the class I error probability, expressed by α, with the two-sided 0. 05 level is the most commonly used. For the case of one-sided α = 0. 025, and the case of α = 0. 025 for the equivalence or non-inferiority test, the essence is still the test level of 0. 05. But in some cases test the setting

It would be something different. For example, to control the overall type I error probability α, the nominal test level α for each test involves multiple tests (e. g., defining multiple main indicators)*Will be less than or equal to α; consider for the interim analysis

α Consumption, and the α for each test*Will be less than the α. Furthermore, for bioequivalence tests, habituation takes a two-sided α of 0. 1.

(C)1994-2022 China Acad■mic Journal Electronic Publishing House. All rights reserved. http://www.cnki.net

Test efficacy: expressed with 1- β, β represents class II error probability. Test efficacy is based on the set α，null hypothesisH_0Is false and the test result is rejectedH_0The probability of。The higher the test efficiency，The greater the probability of finding the difference，However, the required sample size is also larger。In clinical trials，Test efficacy should usually not be less than 80%. In the sample size estimation process, different sample size schemes can be provided through the sensitivity analysis of the test efficacy，For the researchers to choose from。

One-sided and two-sided tests were performed：The sample size for the one-sided test will be significantly smaller than that for the two-sided test 。generally speaking，Statistical testing in the field of medical research routinely uses two-sided tests，If a one-sided test is used，Need to give a charge, sufficient reason。Need to point out，For the general test level of 0. 05, if the unilateral level is 0. 025, the essence is still 0. 05 level.

Balance or non-balanced design：The so-called balanced design，That is, the sample size was the same in each group。When the other conditions remain unchanged，The balanced design efficiency is the highest with the same sample size，That is, the minimum total sample size is required for the test。therefore，The study design should be balanced whenever possible。The nonbalanced design refers to the sample size between the comparison groups，This difference is habitually multiplied，For example, phase III clinical trials of new drugs, because the efficacy of placebo-controlled drugs is relatively certain，Also, for ethical considerations，The sample size of the placebo control group will be smaller，The sample size of the test group was relatively large，Like 2 or 3 times the control group.

(C)1994-2022 China Acad■mic Journal Electronic Publishing House. All rights reserved. http://www.cnki.net

Principle and method of sample size estimation

1. Principles of sample size estimation

The method of sample size estimation is usually calculated from the test statistics formula

Turn it back。in a general way，The statistically inferred effect size can be considered as a function of f (θ) given the model parameter θ, and T represents an unbiased estimated statistic of f (θ)，If the data are derived from a normal distribution，Or according to the central limit theorem, there is

$$\frac{T - f(\theta)}{\sqrt{\hat{Var}(T)}} \sim N(0, 1) \quad (1)$$

Where Var (T) is the variance of the statistic T.

The test level is α, the test efficiency is 1- β, and the two-sided test is required。

In H_0 Under the assumptions, f (θ) = 0, test H_0 at the α level The bound value of the value is

$$Z_{1-\beta}\sqrt{\hat{Var}(T)} = d - Z_{1-\alpha/2} \quad (2)$$

In H_1 Under the assumption，f (θ) = d ，a surname ε= ， $\frac{d}{\sqrt{Var(T)}}$ have

$$1 - \beta = P\{|N(\varepsilon, 1)| > Z_{1-\alpha/2}\} \approx P\{N(\varepsilon, 1) > Z_{1-\alpha/2}\} = P\{N(0, 1) > Z_{1-\alpha/2} - \varepsilon\} = \Phi(\varepsilon - Z_{1-\alpha/2}) \quad (3)$$

Further obtain

$$\hat{Var}(T) = d^2/(Z_{1-\beta} + Z_{1-\alpha/2})^2 \quad (4)$$

More generally, Var (T) can be expressed as a function of the sample size，The sample size was obtained accordingly。The comparison of the means of two groups in normally distributed data is taken as an example，Explain the application of the above principle。

The difference between the two different means is $T = \bar{X}_E - \bar{X}_C$, The difference between the two overall means is found in $\delta = \mu_E - \mu_C$, The two population variances are the same, as σ^2, Then

$$Var(T) = \frac{\sigma^2}{n_E} + \frac{\sigma^2}{n_C} = \frac{r+1}{r}\cdot\frac{\sigma^2}{n_C} \quad (5)$$

Where, r is the ratio of the sample size of the two groups, $n_E = r \cdot n_C$. Put formula (5) into formula (4), yes

$$\frac{\delta^2}{(Z_{1-\beta} + Z_{1-\alpha/2})^2} = \frac{r+1}{r\,n_C}\sigma^2$$

$$n_C = \frac{r+1}{r} \cdot \frac{(Z_{1-\beta} + Z_{1-\alpha/2})^2 \sigma^2}{\delta^2} \quad (6)$$

The above derivation is based on the overall variance σ^2 Known situation.equal σ^2 When unknown, we use the sample variance S^2 As its estimate, the test statistic follows t distribution，Then, as in the above formula Z(.) Need to use t(.) Replace the value.

Based on a normal distribution，have

$$1 - \beta = \Phi\left(\sqrt{\frac{r n_E \delta^2}{(r+1)\sigma^2}} - Z_{1-\alpha/2}\right) \quad (7)$$

Based on the t distribution, the test statistic follows the non-central t distribution, and the sample size can be obtained by iterative calculation，approach

$$\sigma^2(\lambda_i, \eta_i, \gamma_i) = \lambda_i^2\left[\frac{\lambda_i}{\lambda_i + \eta_i} + \frac{\lambda_i \gamma_i e^{-(\lambda_i+\eta_i)T T}(1 - e^{(\lambda_i+\eta_i-\gamma_i)AT})}{(\lambda_i + \eta_i)(\lambda_i + \eta_i - \gamma_i)(1 - e^{-\gamma_i AT})}\right]^{-1},$$

$$i = E, C \quad (11)$$

When the statistic T = logHR, we can also first find Var (T), and then calculate the sample size in formula (4). At this time, the obtained sample size calculation formula can be disassembled into a number of required positive events (number of events) And the expression of the probability of individual positive events, which is also the most common method。

2. Sample size estimation method

Sample size estimation method，That is, the formula for calculating the sample size，Should

ca ■ ec ro se. reserve . .cn

$1 - \beta = probt(t_{1-\alpha/2}, n_E(r+1)-2, n_E(r+1)-2, \lambda\sqrt{\frac{m_A\delta^2}{(r+1)\sigma^2}})$ (8)

The sample size estimate for survival analysis is, although special，But the same principle applies。Using survival analysis data based on the exponential distribution assumption, the case enrollment time (accrual period) is AT

The full length (total time) was TT and the follow-up time (follow-up period) was TT-AT. Suppose group i j th patient survival time t_{ij}The compliance risk rate (hazard rate) is λ_iOf the exponential distribution, the group i lost follow-up rate follows the risk rate η_iThe exponential distribution of，Enrollment timez_{ij}Following an exponential distribution truncated at AT with a probability density function of $g(z) = \gamma e^{-\gamma z} / (1 - \gamma e^{-\gamma AT})$, $0 \le z \le AT$, In the formula，$\gamma > 0$Indicate early entry Group has more cases; $\gamma < 0$ indicates more later enrolled cases; $\gamma = 0$ is generally defined as $g(z) = 1 / AT$, indicating uniform patient enrollment. The follow-up time for each individual is given at x_{ij}And status ζ_{ij}It can be expressed as: $(x_{ij}, \zeta_{ij}) = (\min(t_{ij}, TT - z_{ij}), I\{t_{ij} \le TT - z_{ij}\})$, where $\zeta = 1$ indicates a positive event and $\zeta = 0$ indicates a censored event.

The difference between the risk rate of the test group and the control group is $T = \hat{\lambda}_E - \hat{\lambda}_C$，

The difference in the risk rate of the corresponding population is $\delta_\lambda = \lambda_E - \lambda_C$，Is

$$Var(T) = \frac{\sigma^2(\lambda_E)}{n_E} + \frac{\sigma^2(\lambda_C)}{n_C} \quad (9)$$

Put formula (9) into formula (4) and order $n_E = r \cdot n_C$，n_EAnd n_CThe sample size of the test and control groups, respectively, and r is the test group and pair

Proportion of the sample size，There are

$$\frac{\sigma^2(\lambda_E)}{n_E} + \frac{\sigma^2(\lambda_C)}{n_C} = \delta_\lambda^2 / (Z_{1-\beta} + Z_{1-\alpha/2})^2$$

$$n_C = \frac{(Z_{1-\beta} + Z_{1-\alpha/2})^2 \sigma^2}{\delta_\lambda^2} \left(\frac{\sigma^2(\lambda_E)}{r} + \sigma^2(\lambda_C) \right) \quad (10)$$

(C)1994-2022 China A d mic Journal El t nic Publishing Hou All rights d http://www ki.net

Chinese Journal of Health Statistics , Aug . 2015, Vol . 32, No. 4

Wei source, through examples to illustrate, but also gives the SAS 9.2 software implementation program, easy for the application of readers. Table 1 lists the common usage

The sample size estimation method of the，It can be regarded as an index of the sample size estimation method by statistical test method。

Table 1 Index of common sample size estimation methods in clinical trials

statistical analysis technique	Type of group number test		Statistical tests corresponding to the sample size estimation method
Mean comparison or nonparametric technique	single unit	difference examination	one follow white repeat repeated survey measure basea surname G ree n h ou se One-sample t-test Paired t-test based on square difference component divide - Ge i ss er Corrected for a one-way repeated-measures ANOVA
		Equivalent / Validity test	Paired t-test for equivalence
	two sets	difference examination	Two-sample t-test (homogeneity of variance) Satterthw aite's t-test (uneven variance) Two-sample t-test based on the log-normal distribution Two-sample t-test based on the ratio Wilcoxon / Mann-Whitne y Rank sum test (continuous variable) Wilcoxon / Mann-Whitney Rank sum test (rank variable) a surname G r ee nh ou se
		Equipotency test	Two-sided equivalence test for parallel group design Two-sided equivalence test for the crossover design Equivalence test based on odds cross-design (continuous variables) Equivalence test based on ratio parallel Equivalence test based on odds cross -group design (rank variables)
	multi group	— difference examination	basea surname Linenature One - w ay ANOVA T w o - w a assemble compare join in marriage y AN O More Compared with Of the one-way
Rate comparison	single unit	difference examination	Exact probability test for the one-sample rate M cNemar χ^2 checkout join in marriage To set upcountbase join in marriage To set upcountbase
		Equipotency test	Equivalence test of Double one-sided equivalence test (test group-control group)
	two sets	difference examination	Two-sample rate comparison Pearson χ^2 checkout The Pearson χ for the two-sample rate comparisons^2 Test (calculate one of the sample rate) limitand so oncheck up tonelimitand so oncheck up The Yetes-corrected χ for the two-sample rate comparison^2 Test (calculate sample size or test efficacy) The Yetes-corrected χ for the two-sample rate comparison^2 Test (calculate one of the sample rate) based on Stoked in test class effect two one try examine group - To illuminate try examine group - To illuminate Fisher, Exact probability test two component class Construction of ratio test of two groups More group construct CMH checkout Corrected for
		Equipotency test	Equivalence test for the two-sample rate comparison Test based on non-inferiority limit (test group-control group) two group finger count component cloth two group finger count component cloth
	Multiple groups- -difference test		Multiple sample-rate comparisons of the χ^2 checkout
survival analysis	Test of ——— difference between the two groups		The log-ran k test for comparing the two group survival curves (specific correspond to gene and so on examine group - nature To illuminate Trend test Compared with)
correlation analysis	One-group ——— difference test		According to the group and so on join count with and so on mistake bad examine nature checkout

ca ec ro se. reserve . .cn

Other considerations for the sample size estimation

1. Adjustment of the sample size

The sample size estimated by statistical methods is the minimum sample size required to meet a clinical trial under a given condition 。During the actual test process，Due to the shedding and elimination of the cases 、Poor case compliance and other reasons，This causes a decrease in the number of evaluable cases。therefore，Moderate sample size based on the sample size estimation，To ensure that the final effective sample size can be met Requirements for a minimum sample size。From an analytical point of view，The final evaluable sample size (i. e. meeting the protocol dataset (per-protocol set) shall be guaranteed

(C)1994-2022 China A d mic Journal El t nic Publishing Hou All rights d http://www ki.net

Number of cases) should be greater than the sample size obtained by the sample size estimation method. Sample size adjustment usually considers the shedding rate of no more than 20%, and how to determine the specific shedding rate，Will depend on the different research projects，The basis for the determination mainly comes from the professional judgment，Or by the meta-analysis of previous research data as an important reference.

When the results of the subgroup analysis are the primary efficacy measure，The final subgroup of evaluable cases should reach a minimum sample size。

The results of clinical trials may be subject to certain prognostic factors (covariates) influence，Such as age、sex、The degree of illness, etc。Prognostic factors are generally not considered when estimsample size，Mainly because randomization balances the covariates between the groups。

2. Reestimate of the sample size

The sample size is more used for adaptive design。The three commonly used methods to reestimate the adaptive design sample size are as follows。

(1) Sequential design in groups: the sample size of each group is fixed, and the purpose of each interim analysis is to make a decision on whether to terminate the trial (success or failure) or the trial entering the next cycle。

(2) Fixed interim analysis: each interim analysis，Make new estimates and adjustments of the sample size accordingly，But the test hypothesis is not tested。

(3) The combination of the above two methods: each interim analysis both re-estimates the parameters，Make new estimates and adjustments to the sample size, and test the hypothesis to terminate the test (successful or failed) or enter the next cycle.

All three methods belong to the sample size estimation problem for mid-term analysis, and detailed procedures can be found in the literature [9]. It should be noted that sample size reestimation based on interim analysis should be blinded as far as possible。

Requirements for sample size estimation in the clinical trial protocol

In the clinical trial protocol，A clear and complete elaboration of the sample size estimates is needed，Should include at least the following content：Study hypothesis of the trial，The type of control，Compare the type，Design model，Main indicators，Statistical analysis methods to be used, the source and basis of parameters (including equivalent or non-inferiority thresholds), test level (if multiple tests or mid-term analysis is involved, the basis for determining the test level should be explained), test efficiency, single and double
Side test, allocation proportion, sample size estimation method and its source (column references), the software used and its version, sample size adjustment and its basis, each
Sample size allocation (list) of groups and center, specified specified if entry。

FAQ

Q 1. What is the dedicated software for sample size estimation?

Current common sample size estimation software are nQueryAdvisor + nTerim, PASS, DSTPLAN, G * Pow er, PC-Size, PS, SAS Pow er and Sample Size application (PSS), Stata, R 。Of these software, nQuery and PAS S are the most commonly used, and they cover almost all sample size statistical methods。Below are soft on these

(C)1994-2022 China Acad■mic Journal Electronic Publishing House. All rights reserved. http://www.cnki.net

Make a brief introduction。

nQuery Advisor + nTerim: Irish Statistical Solutions, a commercial software developed by the company, consists of nQuery Advisor 7 software and nTerim module. The former was originally an independent sample size estimation software，The latter is the sample size estimation module specifically for interim analysis。 The latest version is 3. 0 and runs on the Window s platform. The software, also recognized by the FDA, European Medicines Agency, Japan and South Korea, is used by 49 of the world's top 50 pharmaceutical companies and biotechnology companies. The content has almost covered the sample size calculation There are aspects. A series of Chinese Health Statistics series systematically introduces common sample size estimation methods[9], The calculation formula and its provenance are given, along with the operation main interface of sample size estimation professional software nQuery Advisor 7. 0 and the interface of parameter setting in sample size estimation，at the same time The SAS 9.2 software implementation program is also provided for the convenience of the general readers.

PASS: NCSS, developed by the company, is a commercial software running under the Window s platform, and the latest version is 13. Similar to nQuery, it also covers almost all sample size calculation methods, and its official website claims to use more than 230 statistical methods.

DSTPLAN: It is a free software running in the Window s environment. It is itself based on the Fortran language and developed by Anderson Cancer Center. Statistical analysis methods included were t-test, correlation analysis, comparison of rates, contingency table tests for 2 N, and differential test for survival analysis。

G * Pow er: It is a free software that runs in Window s and M ac OS X environments，Developed by the University of Dusseldorf, Germany。The statistical analysis methods included are t-test, One-w ay ANOV A, and regression analysis、Correlation analysis as well as the goodness-of-fit analysis。The software gives the effect size immediately after the user enters the key parameters。

PC-Size: It is a free DOS command-line software running in the Window s environment. The statistical analysis methods included are t-test, analysis of variance 、regression analysis、Correlation analysis as well as a comparison of the rates。The software can also calculate the effect sizes。

PS: It is a free software running in Wind o w s environment, including statistical analysis methods including t-test, chi-square test and Fisher Probability method, M cNemar-test, regression analysis, and survival analysis.

SAS / Pow er and Sample Size application (PSS): This software runs in a Window s environment and is installed throughout the entire SAS family. Although developed by the SAS company, the statistical analysis methods included were very limited, with only the t-test, comparison of rates, correlation analysis, and regression analysis、Analysis of variance as well as the survival analysis。

Stata / R: Stata and R should strictly be a programming language rather than off-the-shelf software。In theory, just programmed properly，Statistical methods for any sample size calculation can be implemented。

besides，There is also a large number of online sample size estimation software or programs，only It is difficult to comment on its correctness and authority，So encourage it (go down to page 733)

(C)1994-2022 China Acad■mic Journal Electronic Publishing House. All rights reserved. http://www.cnki.net

Malignancies, injuries and poisoning, and circulatory diseases are the three main causes of death in middle-aged people, and the number of deaths in men is significantly higher than in women.the author holds that, First, indeed strengthen environmental protection; second, the whole society should care for middle-aged people from the perspective of society, psychology and physiology, Make the middle-aged people have a healthy body and mind and good living habits, Reduce the occurrence of diseases; third, strengthen the training of labor safety and driving safety and the implementation of relevant safety system, Safety is more important than Mount Tai, Reduce death from injury and poisoning; fourth, medical personnel should pay attention to health education, A stitch in time saves nine, Efforts to achieve the early detection, early diagnosis and early treatment of diseases such as malignant tumors, Improve the quality of health care, Prolonged survival period, Reduce mortality rates.

reference documentation

[1] Huang Yu. Discussion of nursing intervention in middle-aged hypertensive patients. The Chinese ethnic minorities and folk medicine Medicine, 2010,19 (15): 221.

[2], Yang Guangfu, Wang Yuzhang. Prevention of stroke ——— Risk stratification and adjustment of middle-aged and elderly patients rule. Medical Research and Education, 2010,27 (2): 54-59.

[3] Wang Feng, Deng Bing. Sample survey of health status and living quality of middle-aged people in Guiyang community. Guiyang Medicine, 2010,34 (2): 169-170.

[4] Ni Ruli. Analysis of the cause of death in elderly inpatients. Chinese Hospital Statistics, 2008,15 (2): 128-129.

[5], Du Chunhua. Cause of death in a hospital from 2005 to 2009. Chinese medical Pharmaceutical Guidelines, 2010,8 (19): 62-64.

[6] Statistical Bulletin on the development of health undertakings in China. Health Statistics Information Center, 2007-05-09.

[7], Fan Xiaoxin, Cao Xia. Statistical analysis of hospitalized deaths aged 45 to 59. The Chinese medical case, 2010, 11 (8) : 31.

[8], Zhou Xiaolan, Shi Lei. Characteristics of 3030 inpatient deaths from 2007 to 2011. West China Medicine, 2012,27 (7): 1032-1035.

[9] Yuan Xueyu. Analysis of deaths of middle-aged and elderly inpatients from 1993 to 2011. Translational medicine Magazine, 2012,1 (2): 97-99.

(Editor in charge: Deng Yan)

(Page. 731 above.)

Use more authoritative nQuery and other PA SS software, can also be based on rights The source of the estimation method is implemented by own programming。

Q 2. Under what circumstances can the sample size estimation not be made?

For confirmatory clinical trials，All require sample size estimation。No sample size estimation can be made for pre-trials and exploratory trials (such as phase II of new drugs) ，However, it is necessary to explain the reason of no sample size estimation cause。If a standard specifies the minimum number of test cases，You can also not do it Sample size estimates。

Q 3. What is the significance of calculating the test efficiency based on the sample information? Test efficacy is expressed as a 1- β, where β is the class II error probability.

As is known from the statistical theory，In cases where the overall distribution determined by the parameters is known，To calculate the β；And the sample information can not reflect the overall parameters，Therefore, the β cannot be calculated based on the sample information，Nor can the true test efficiency be calculated。therefore，inferring the magnitude of test efficacy based on sample information is unreliable，Should avoid；But if it is for further research Providing clues is a reference。

Q 4. Is sample size estimation required for subgroup analysis?

Whether the subgroup analysis needs to estimate the sample size should be determined according to the purpose of the study。If the purpose of the study is to confirm the conclusions of a subgroup，For example, one of the purposes of the phase III clinical trial of a new drug is to confirm the effectiveness of the drug in the Chinese mainland population，Then

ca ■ ec ro se. reserve . .cn

the study protocol in addition to estimate the overall sample size，Sample size estimation is also required specifically for this subgroup of the mainland Chinese population。If the conclusions of a subgroup or several subgroups are not confirmed，No sample size estimation is required for the subgroup analysis 。in this case，Even though the data processing results showed statistical significance in the subgroup analysis，It should not be regarded as a confirmatory conclusion for subgroups。See corresponding statistical considerations for CCTS.

What is the difference in the estimated sample size?

The minimum sample size required by the registration administration method is mainly based on the consideration of safety evaluation，However, the estimated sample size based on statistical considerations is mainly to meet the needs of effectiveness evaluation。The sample sizes determined from the above two aspects are most likely different ，The final sample size should be the larger ones 。

reference documentation

[1] ICH E 10 : Choice of Control Group and Related Issues in Clinical Trials . US , Department of Health and Human Services Food and Drug Administration , 2001 .

[2] ICH E 9 : Statistical Principles for Clinical Trials. US , Department of Health and Human Services Food and Drug Administration , 1998 .

[3] State Food and Drug Administration Good Clinical Practice since 2003 It will take effect on September 1

[4] CCTS working Group, written by Wang Tong and Yi Dong. Statistics of multiplicity problems in clinical trials think over. Health Statistics of China, 2012,29 (3): 445-450

[5] Julious SA. Sample Sizes for Clinical Trials. Boca Raton : Chapman & Hall / CRC , 2010 .

[6] M achin D , Campbell MJ , Tan SB , et al. Sample Size Tables for Clin i - cal Studies. UK : Wiley -Blackw ell 3rd edi t , 2009 .

[7] Ryan TP. Sample Size Determination and Pow er. USA : John Wiley & Sons , Inc , 2013 .

[8] Chow SC. Sample Size Calculations in Clinical Research . U S A : Chapman & Hall / CRC 2nd Edit , 2008 .

[9] Chen Bingyan. Sample size estimation and its implementation on nQuery and SAS software (Series theory Article 19). China Health Statistics, 2012,29 (1) -2014; 31 (2).

[10] Elashoff JD. nQuery Advisor User' s Guide. Ireland : Statistical Solu- tions Ltd . 2007 .

[11] SAS Institute , Inc. Getting Started w ith the SAS Pow er and Sample Size Application . North Carolina : SAS Institute , Inc. 2004 .

[12] Crisp A , Curtis P. Sample size estimation for non-inferiority trials o f time-to -event data. Pharmaceutical statistics , 2008, 7 :236-244.

(Editor in charge: Deng Yan)

Q 5. Minimum sample size and statistics stipulated by the registration measures

(C)1994-2022 China A d mic Journal El t nic Publishing Hou All rights d http://www ki.net
